# Forced vital capacity trajectories in patients with idiopathic pulmonary fibrosis: a secondary analysis of a multicentre, prospective, observational cohort

**DOI:** 10.1016/S2589-7500(22)00173-X

**Published:** 2022-11-01

**Authors:** Hernan P Fainberg, Justin M Oldham, Philip L Molyneaux, Richard J Allen, Luke M Kraven, William A Fahy, Joanne Porte, Rebecca Braybrooke, Gauri Saini, Morten A Karsdal, Diane J Leeming, Jannie M B Sand, Isaac Triguero, Eunice Oballa, Athol U Wells, Elisabetta Renzoni, Louise V Wain, Imre Noth, Toby M Maher, Iain D Stewart, R Gisli Jenkins

**Affiliations:** National Heart and Lung Institute, Imperial College London, London, UK; Division of Pulmonary and Critical Care Medicine, University of Michigan, Ann Arbor, MI, USA; National Heart and Lung Institute, Imperial College London, London, UK; Royal Brompton and Harefield Hospitals, Guy’s and St Thomas’ NHS Foundation Trust, London, UK; Department of Health Sciences, University of Leicester, Leicester, UK; Department of Health Sciences, University of Leicester, Leicester, UK; Discovery Medicine, GlaxoSmithKline Medicines Research Centre, Stevenage, UK; Nottingham Respiratory Research Unit, NIHR Biomedical Research Centre, University of Nottingham, Nottingham, UK; Nottingham Respiratory Research Unit, NIHR Biomedical Research Centre, University of Nottingham, Nottingham, UK; Nottingham Respiratory Research Unit, NIHR Biomedical Research Centre, University of Nottingham, Nottingham, UK; Nordic Bioscience, Herlev, Denmark; Nordic Bioscience, Herlev, Denmark; Nordic Bioscience, Herlev, Denmark; Computational Optimisation and Learning Lab, School of Computer Science, University of Nottingham, Nottingham, UK; DaSCI Andalusian Institute in Data Science and Computational Intelligence, University of Granada, Granada, Spain; Discovery Medicine, GlaxoSmithKline Medicines Research Centre, Stevenage, UK; National Heart and Lung Institute, Imperial College London, London, UK; Royal Brompton and Harefield Hospitals, Guy’s and St Thomas’ NHS Foundation Trust, London, UK; National Heart and Lung Institute, Imperial College London, London, UK; Royal Brompton and Harefield Hospitals, Guy’s and St Thomas’ NHS Foundation Trust, London, UK; Department of Health Sciences, University of Leicester, Leicester, UK; National Institute for Health Research, Leicester Respiratory Biomedical Research Centre, Glenfield Hospital, Leicester, UK; Pulmonary and Critical Care Medicine, University of Virginia, Charlottesville, VA, USA; National Heart and Lung Institute, Imperial College London, London, UK; Royal Brompton and Harefield Hospitals, Guy’s and St Thomas’ NHS Foundation Trust, London, UK; Keck School of Medicine, University of Southern California, Los Angeles, CA, USA; National Heart and Lung Institute, Imperial College London, London, UK; National Heart and Lung Institute, Imperial College London, London, UK; Royal Brompton and Harefield Hospitals, Guy’s and St Thomas’ NHS Foundation Trust, London, UK

## Abstract

**Background:**

Idiopathic pulmonary fibrosis is a progressive fibrotic lung disease with a variable clinical trajectory. Decline in forced vital capacity (FVC) is the main indicator of progression; however, missingness prevents long-term analysis of patterns in lung function. We aimed to identify distinct clusters of lung function trajectory among patients with idiopathic pulmonary fibrosis using machine learning techniques.

**Methods:**

We did a secondary analysis of longitudinal data on FVC collected from a cohort of patients with idiopathic pulmonary fibrosis from the PROFILE study; a multicentre, prospective, observational cohort study. We evaluated the imputation performance of conventional and machine learning techniques to impute missing data and then analysed the fully imputed dataset by unsupervised clustering using self-organising maps. We compared anthropometric features, genomic associations, serum biomarkers, and clinical outcomes between clusters. We also performed a replication of the analysis on data from a cohort of patients with idiopathic pulmonary fibrosis from an independent dataset, obtained from the Chicago Consortium.

**Findings:**

415 (71%) of 581 participants recruited into the PROFILE study were eligible for further analysis. An unsupervised machine learning algorithm had the lowest imputation error among tested methods, and self-organising maps identified four distinct clusters (1–4), which was confirmed by sensitivity analysis. Cluster 1 comprised 140 (34%) participants and was associated with a disease trajectory showing a linear decline in FVC over 3 years. Cluster 2 comprised 100 (24%) participants and was associated with a trajectory showing an initial improvement in FVC before subsequently decreasing. Cluster 3 comprised 113 (27%) participants and was associated with a trajectory showing an initial decline in FVC before subsequent stabilisation. Cluster 4 comprised 62 (15%) participants and was associated with a trajectory showing stable lung function. Median survival was shortest in cluster 1 (2·87 years [IQR 2·29–3·40]) and cluster 3 (2·23 years [1·75–3·84]), followed by cluster 2 (4·74 years [3·96–5·73]), and was longest in cluster 4 (5·56 years [5·18–6·62]). Baseline FEV_1_ to FVC ratio and concentrations of the biomarker SP-D were significantly higher in clusters 1 and 3. Similar lung function clusters with some shared anthropometric features were identified in the replication cohort.

**Interpretation:**

Using a data-driven unsupervised approach, we identified four clusters of lung function trajectory with distinct clinical and biochemical features. Enriching or stratifying longitudinal spirometric data into clusters might optimise evaluation of intervention efficacy during clinical trials and patient management.

**Funding:**

National Institute for Health and Care Research, Medical Research Council, and GlaxoSmithKline.

## Introduction

Idiopathic pulmonary fibrosis is a chronic respiratory disease, characterised by progressive lung scarring and loss of lung function.^1^ The prognosis is poor, with a median survival of 3–5 years.^2^ However, the progression of disease is variable, with some patients showing stable lung function over time, whereas others progress rapidly or experience episodes of acute deterioration.^2,3^ Change in forced vital capacity (FVC) is an accepted marker of disease progression in patients with idiopathic pulmonary fibrosis.^4–7^ Identifying and characterising pulmonary function trajectories soon after diagnosis^3^ is crucial for establishing prognosis, making clinical management decisions,^2,3,6,7^ and interpreting results from interventional clinical trials.^2,6,7^

Evaluation of disease progression in clinical trials and observational studies in patients with idiopathic pulmonary fibrosis is often hampered by missing data on lung function,^6–9^ affecting the power and accuracy of statistical models for assessing decline of lung function.^4,5,10,11^ As idiopathic pulmonary fibrosis progresses, missed spirometry visits promote survivor bias by raising the mean FVC because missing values are associated with exacerbation of the condition or mortality among patients.^6–8^ To mitigate this bias, previous studies have used various methods to adjust for data loss.^4–7,10,11^ However, these approaches can introduce alternative biases, making it difficult to accurately measure and model the disease trajectory of idiopathic pulmonary fibrosis over extended time periods.^6–9,12,13^

Machine learning algorithms can overcome some assumptions and might mitigate biases induced by other imputation methods.^14,15^ Missing data remain an issue for machine learning tools; however, additional mathematical techniques can estimate numerous possible outcomes by resampling the underlying distributions thousands of times^14,15^ to generate enhanced synthetic datasets, which can be used to train machine learning algorithms^16^ to operate as an imputation tool.^17,18^

We aimed to enhance the power of a longitudinal cohort of patients with incident idiopathic pulmonary fibrosis through imputation of data on lung function to estimate FVC loss due to disease progression. Subsequently, we applied unsupervised self-organising maps (SOMs) to identify distinct clusters of disease trajectories among patients with idiopathic pulmonary fibrosis, which could inform disease management and improve the efficacy of clinical trials.

## Methods

### Study design

We did a secondary analysis of longitudinal data on FVC collected from a cohort of patients with idiopathic pulmonary fibrosis from the PROFILE study;^19^ a multicentre, prospective, observational cohort study. We also performed a replication of the analysis on an independent dataset (ie, the replication cohort), obtained from the Chicago Consortium, which included longitudinal FVC measures obtained from the UUS study (in the USA, the UK, and Spain) collected by the University of Chicago (Chicago, IL, USA).^20^ The PROFILE study and the replication cohort have been described previously (appendix p 1).^19,20^

### Data analysis

Imputation methods were chosen on the basis of the data being continuous rather than categorical, and following literature review.^6,7,13–15,18^ Methods included simple interpolation of missing values, including conventional linear regression, last observation carried forward, and 10% annual reduction in percentage predicted FVC (−10% decline per year),^6,7,13^ as well as machine learning approaches, including random forest^15^ and k-nearest neighbours^14^ classifiers^16^ capable of dealing with nonlinear data and data that are not normally distributed.^14,15,18^ Due to the longitudinal connectivity between the spirometric visits related to a patient, all imputations were performed as consecutive chained equations.^18^

For testing imputation methods, we used the complete dataset, consisting of 82 patients who completed all six spirometric visits (appendix p 13), split into learning datasets (57 [70%]) and test datasets (25 [30%]). Internal ten-fold cross-validation was used to optimise machine learning models. Synthetic simulation of missing data was conducted by removing data randomly from the test dataset, in proportion to the distribution of the occurrence of missing spirometric appointments in the whole PROFILE cohort. The lowest normalised root mean squared deviation (NRMSD) from separate models was used to assess the reliability of imputation. This index is used to measure the differences between values predicted by a model and observed values. The NRMSD represents the square root of the differences between predicted and observed values divided by the SD of the observed values.^14,15^

To minimise survival bias and to increase statistical power, we did an analysis that included imputed values at all timepoints, regardless of the reason for missingness, including death. Based on the results of the complete dataset, we built a continuous autoregressive model.^17^ Integrating this model into Markov Chain Monte Carlo (MCMC) allowed incorporation of stochastic volatility over time, simulating events not experienced by patients in the complete dataset, such as abrupt FVC decreases or below-mean FVC values preceding patient death,^11,17^ which we termed the naive dataset. To mitigate against residual survival bias in this naive dataset, we generated a further theoretical dataset (10 000 simulations each). In this dataset, we substituted 41·7% dummy values (including FVC=0) into the naive dataset and distributed these values proportionally to the mortality rate observed from the first year to the third year in the PROFILE study. We assessed the sensitivity of these imputation approaches by comparing NRMSD values across all spirometric visits.

We performed the unsupervised cluster analysis using SOMs. As a preprocessing step, we normalised the data by centralisation and scaling, which transformed the data into scale-free values. We performed hyperparameter optimisation before clustering. The SOM network was trained for the corresponding dataset for 200 iterations to minimise quantisation error. The learning rates started from 1·00 and was set to 0·90 (ordering) and to 0·02 (tuning), and a neighbourhood distance was set at 1·00 with hexagonal topology.^21^ Due to algorithmic similarities between k-means and SOMs, we used the Elbow method to identify the optimal number of clusters in our datasets. The validity (or stability) of each cluster was assessed by Jaccard indices after the sensitivity analysis. The minimum threshold for cluster stability by Jaccard indices was set at 50%.^21^

We performed three additional sets of sensitivity analyses on the generated clusters. First, clusters were generated by use of 3 years of spirometry data from the following datasets: the complete PROFILE dataset, the complete PROFILE dataset excluding patients with data missing due to death, and data from patients who completed all spirometric visits without imputation. The second sensitivity test analysed the clusters generated by use of spirometry data from baseline to the first year, baseline to the second year, baseline to the third year, and from patients who completed all six spirometric visits. Theses analyses were performed in the same way in the replication cohort. The final sensitivity test included the cluster generation by k-means on the PROFILE dataset.

Serum biomarkers were measured from samples that were prospectively collected at baseline and analysed as previously described (appendix pp 1–2).^19^

### Statistical analysis

We implemented a workflow using open-source packages from the R project (version 4.1.1). Scripts are deposited online. To evaluate associations between lung function and disease trajectory between clusters, we applied a mixed-effects linear model with repeated measures analysis of annual rate of change in FVC. We performed the mortality risk assessment between clusters using hazard ratios (based on the Cox proportional hazards model), Kaplan-Meier plots, and log-rank tests. Survival probability at any particular timepoint was calculated by the formula: ([number of participants living at the start – number of participants who died] / number of participants living at the start).

Estimates for the Cox proportional hazards model and mixed-effects linear model tests were adjusted for covariance and limited to baseline percentile-predicted FVC in all analyses. Wilcoxon’s signed-rank test was used for continuous variables, and Fisher’s exact test was applied for categorical variables.

All comparisons among clusters were adjusted with the Bonferroni correction method. Data are median (95% CI), unless otherwise indicated. All statistical tests were two-sided, and p<0·05 was considered to be significant.

### Role of the funding source

The funder of the study had no role in study design, data collection, data analysis, data interpretation, or writing of the report.

## Results

**Figure 1:**
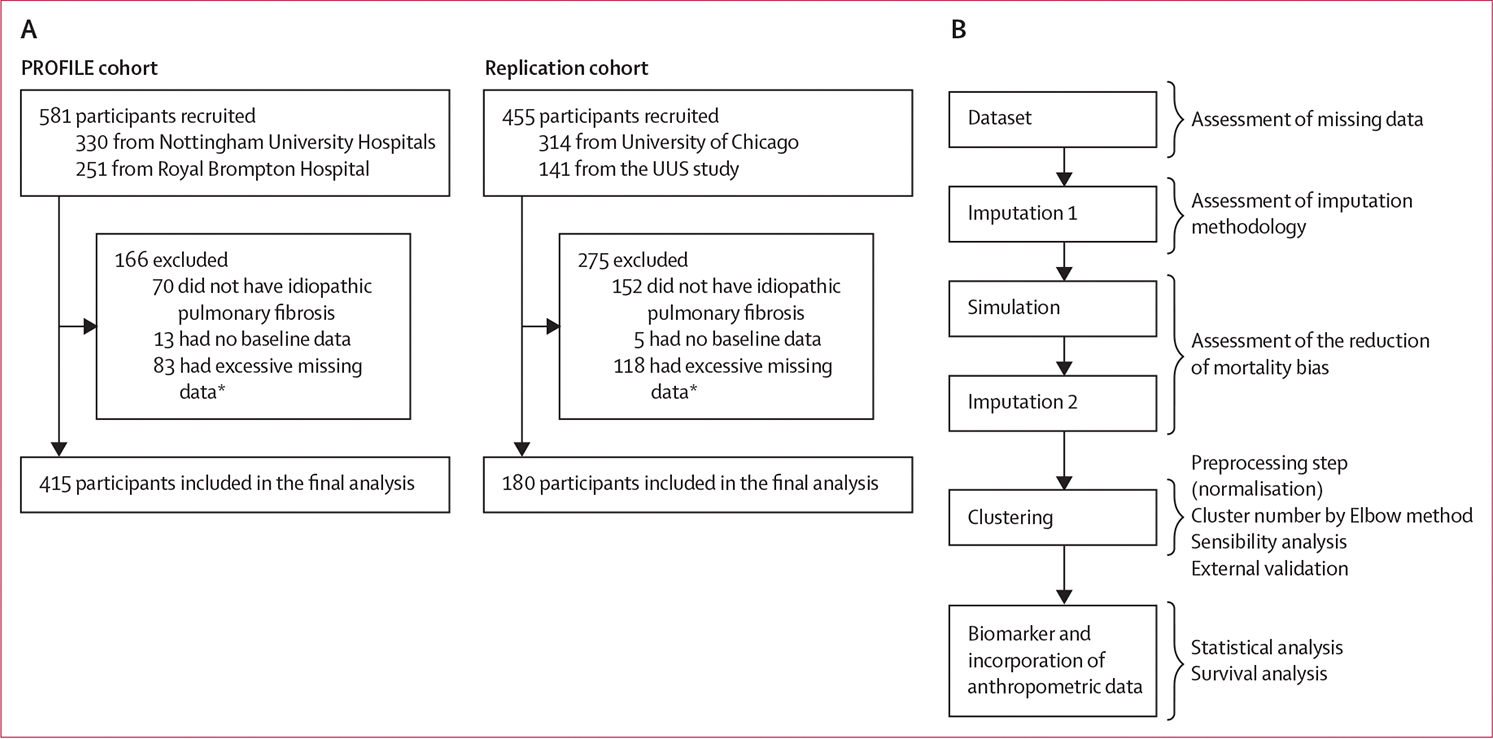
Cohort profiles and data analysis workflow (A) Cohort profiles for the PROFILE cohort and the replication cohort. (B) Workflow used to generate each step of the data analysis. FVC=forced vital capacity. *Participants with insufficient data—ie, patients who missed the initial spirometric appointment and were also missing FVC datapoints at 90 days or 180 days.

415 (71%) of 581 participants recruited into the PROFILE study were eligible for this secondary analysis, while 180 (40%) of 455 participants from the independent dataset were eligible for inclusion in the replication cohort (figure 1). Mean baseline FVC was 80·1% (SD 18·9). 321 (77%) participants were men, 94 (23%) were women, and mean age among participants was 70·6 years (SD 7·8; appendix p 13). Data on complete lung function were available in 82 (20%) participants. Data were missing due to death in 173 (42%) participants, of whom 48 (12%) died during the first year, 68 (16%) in the second year, and 57 (14%) in the third year. These missing data values meant that 488 (29·4%) of 1660 data points required imputation for the full analysis of lung function. A further 196 (11·8%) data points were missing for unknown reasons. Overall, from the complete PROFILE dataset of 415 patients, the dataset excluding patients with data missing due to death comprised 242 patients, and all data points were available from 82 patients who completed all spirometric visits.

**Figure 2:**
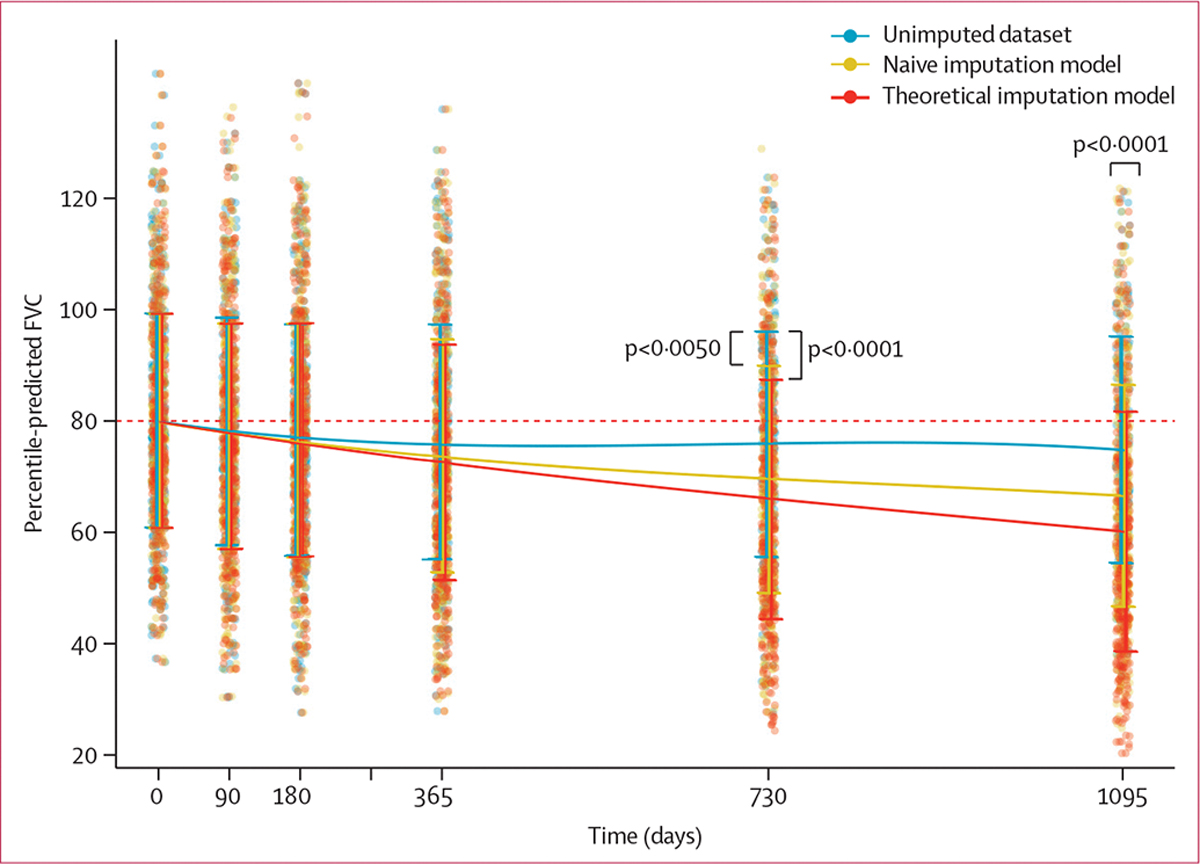
Lung function per imputation model among patients in the PROFILE cohort Each data point represents a single lung function value. Each trendline represents mean (SD) percentile-predicted FVC values for the unimputed dataset, the naive imputation model, and the theoretical imputation model. FVC=forced vital capacity.

Across all simulations, the random forest MCMC approach had the lowest mean NRMSD (0·4 [SD 0·2]), compared with models performing imputation by value interpolation and the other machine learning algorithm, k-nearest neighbours. Linear regression had the highest mean NRMSD (1·3 [0·8]) at each timepoint beyond 6 months, compared with all other methods (appendix p 3). In the sensitivity analysis that was performed for random forest MCMC imputation to compare unimputed data with naive and theoretical imputation models, there was little effect of imputation at 12 months (figure 2). After 2 years, differences were observed between the unimputed dataset (mean value 75·9 [95% CI 73·9–77·8]) and the naive model (69·5 [67·6–71·5]; p=0·0050), and between the unimputed dataset and the theoretical model (66·0 [63·9–68·0]; p<0·0001). After 3 years, differences were observed between all three models (unimputed 74·9 [72·9–76·8] *vs* naive 66·6 [64·7–68·5] *vs* theoretical 60·2 [58·1–62·3]; p<0·0001). The naive random forest imputation model had the lowest NRMSD mean value and was, therefore, used for further SOM cluster analysis (appendix p 4). The cluster sensitivity and validity analyses of these datasets suggested that the optimal number of discrete clusters derived using SOMs across all datasets was four, regardless of the dataset. The mean Jaccard indices, used to assess the validity and internal sensitivity of the clusters obtained, were 0·75 (SD 0·20) for cluster 1, 0·64 (0·16) for cluster 2, 0·73 (0·15) for cluster 3, and 0·63 (0·10) for cluster 4 (appendix pp 5–6, 12).

**Figure 3:**
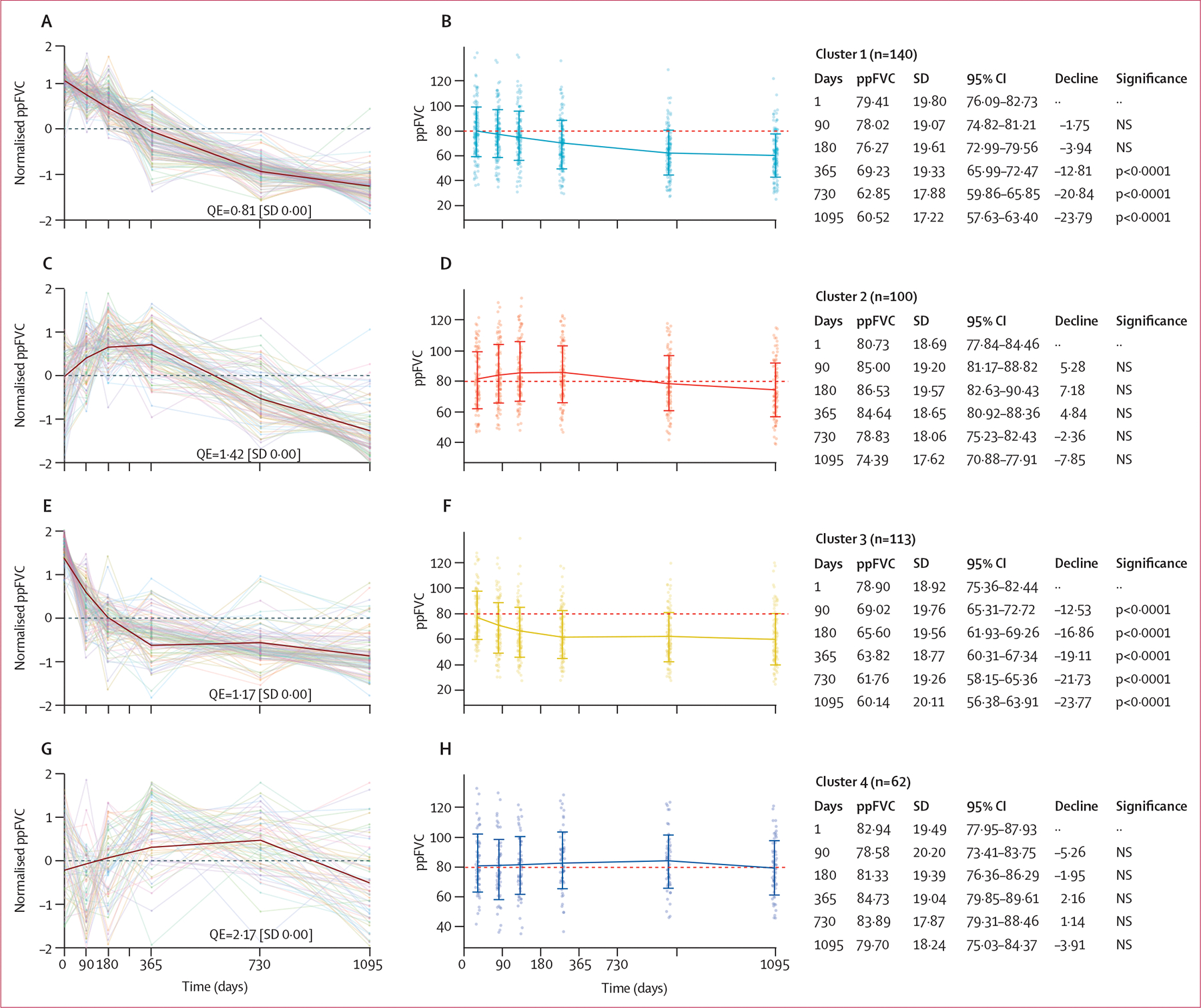
SOMs of FVC trajectory per cluster among patients in the PROFILE cohort Individual data points indicate the values obtained by the naive imputation model for each patient. Trendlines show mean (SD) ppFVC values at each timepoint. Individual spirometry traces clustered by SOMs from each patient are represented as scale-free normalised values for cluster 1 (A), cluster 2 (C), cluster 3 (E), and cluster 4 (G), and as non-normalised values for cluster 1 (B), cluster 2 (D), cluster 3 (F), and cluster 4 (H). Decline refers to percentage year decline in ppFVC from baseline. Significance tested following Bonferroni correction. SOM=self-organising map. FVC=forced vital capacity. pp=percentile-predicted. QE=quantisation error. NS=not significant.

Cluster 1 was the largest cluster, with 140 (34%) of 415 participants, and was associated with a disease trajectory showing a linear decline in mean FVC over 3 years (table 1; figure 3A, B). Cluster 2 comprised 100 (24%) participants and was associated with a trajectory of improving lung function during the first year, before a subsequent decline in function over the second and third year (figure 3C, D). Cluster 3 comprised 113 (27%) participants and was associated with a trajectory of initial linear decline over the first year, before subsequent stabilisation in lung function (figure 3E, F). Cluster 4 was the smallest cluster, comprising 62 (15%) participants, and was associated with a trajectory showing largely stable mean lung function over all 3 years (figure 3G, H).

In the replication cohort, comprising 180 individuals who qualified for imputation, the optimal number of clusters was also four (appendix p 8). SOM analysis showed similar cluster architecture with regard to the size of each cluster and the nature of lung function trajectories, with 74 (44%) participants in cluster 1, 38 (21%) in cluster 2, 42 (23%) in cluster 3, and 26 (14%) in cluster 4 (table 2; appendix p 8). Furthermore, the four clusters generated by SOMs in the PROFILE dataset were reproduced with the k-means clustering algorithm. These k-means clusters had identical architecture and similar membership allocation to those generated by SOMs (appendix p 12).

**Figure 4:**
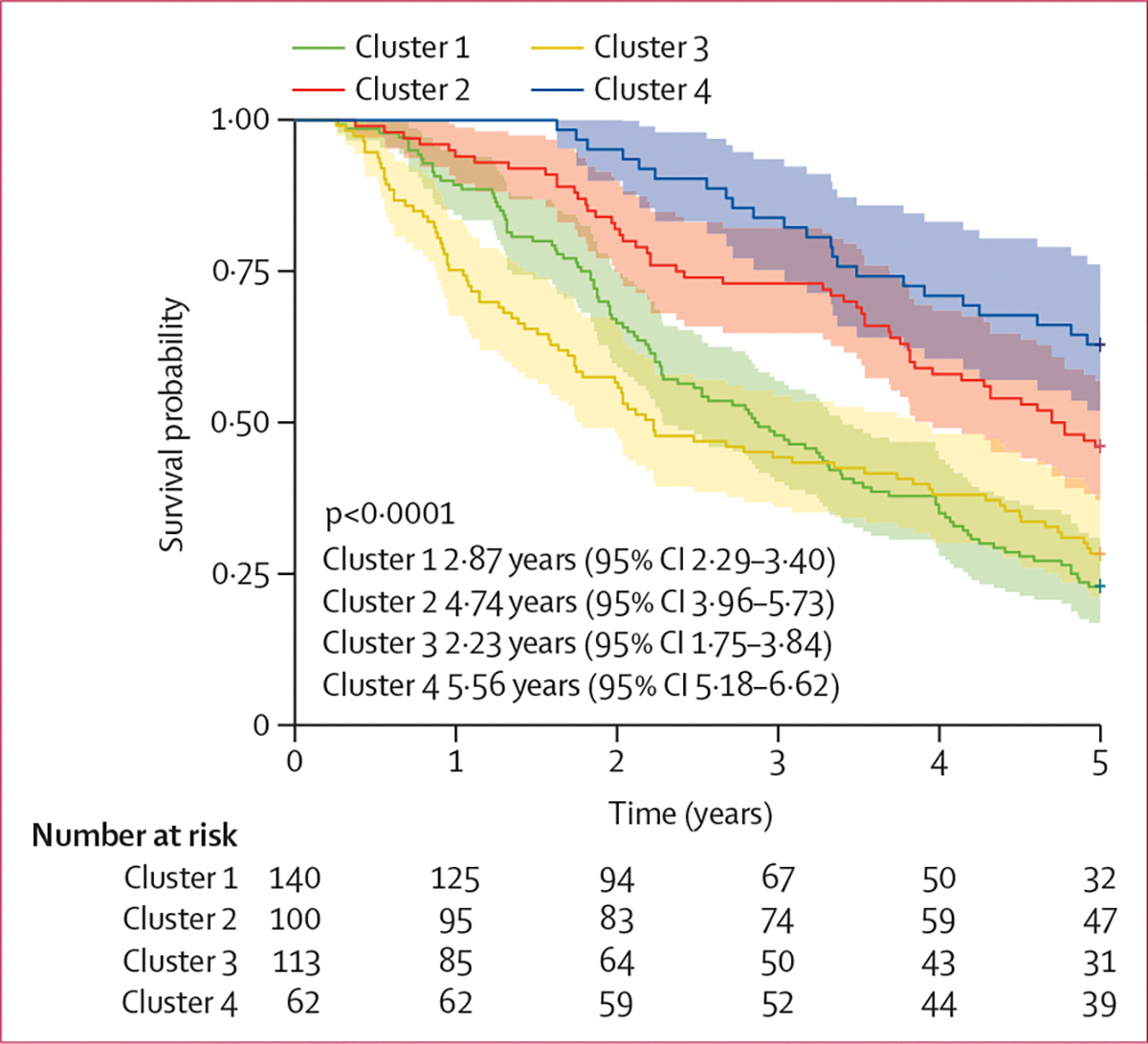
Kaplan-Meier estimates of survival per cluster among patients in the PROFILE and replication cohorts (A) Survival probability estimates (95% CI) based on cluster allocation. 95% CIs were calculated from a non-parametric asymptotic distribution. (B) The number of deaths in every cluster over 5 years.

**Figure 5:**
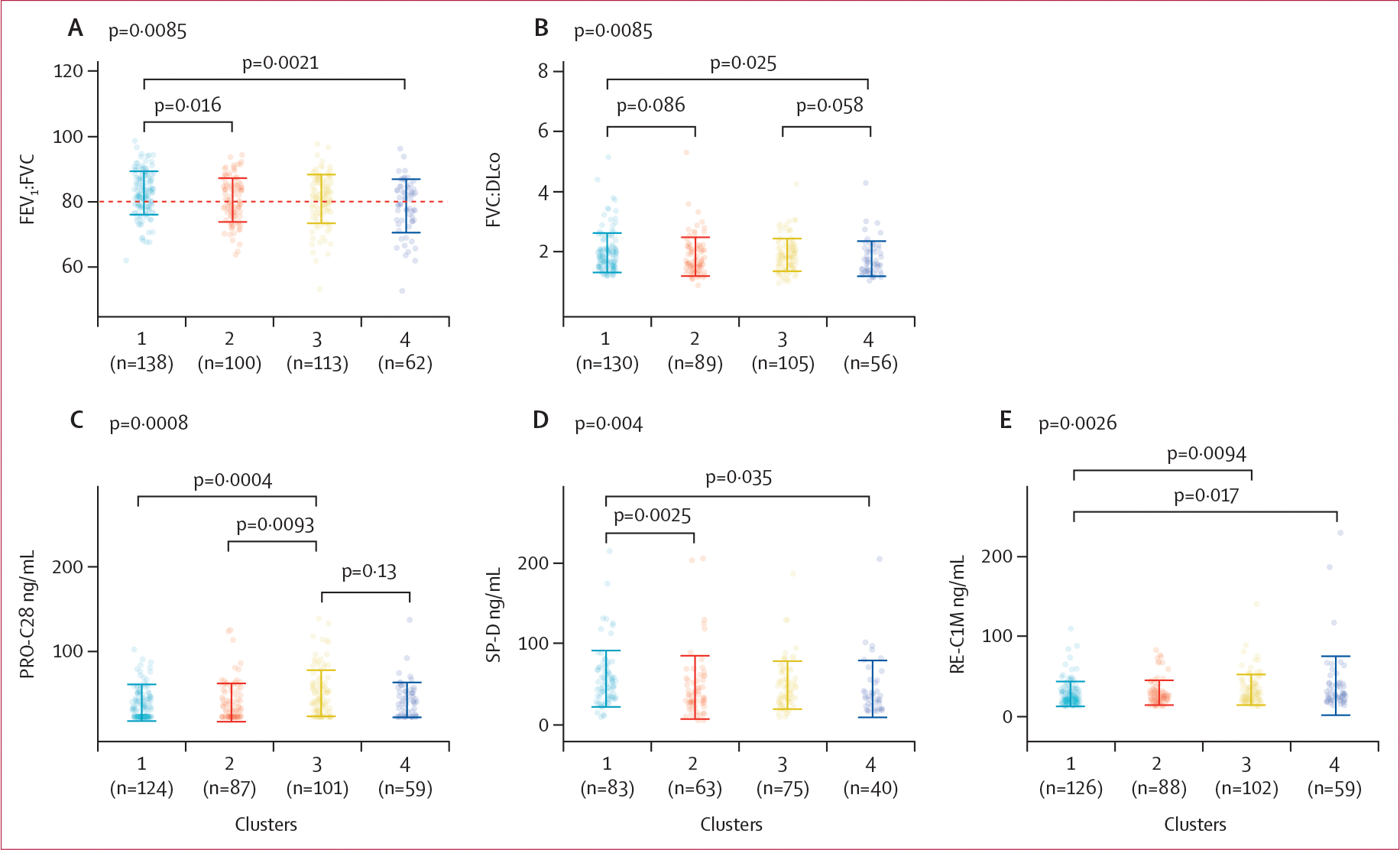
Baseline biochemical characteristics per cluster among patients in the PROFILE cohort Baseline values for FEV_1_ to FVC ratio (A), FVC to DLco ratio (B), and the biomarkers PRO-C28 (C), SP-D (D), and RE-C1M (E) in each cluster obtained from the unimputed dataset. Data are median (IQR); each data point represents an individual value. The overall p values shown represent those obtained from the Kruskal-Wallis test for each biochemical characteristic. Significance was accepted at post-Bonferroni correction p<0·025. FEV_1_=forced expiratory volume in 1 s. FVC=forced vital capacity. DLco=diffusion capacity for carbon monoxide.

Participants in cluster 1 followed a linear decline in lung function, and this represented the most common phenotype in both the PROFILE cohort and the replication cohort (figure 3; table 1; appendix p 8). These patients had similar median survival with and without adjustment for baseline FVC in both cohorts (2·87 years [IQR 2·29–3·40]; figure 4; appendix p 10). In cluster 1, participants were generally younger and contained more never smokers, although the association between smoking status and disease trajectory was not significant (p=0·084). Biochemically, cluster 1 was associated with the highest concentrations of serum surfactant protein-D (SP-D; figure 5).

Cluster 2 was the third most common cluster in both the PROFILE cohort and the replication cohort, and had a low number of never smokers in both cohorts (table 1). This cluster was associated with older age and a history of ever smoking. Concentrations of SP-D, as well as the FEV_1_ to FVC ratio, were significantly lower in cluster 2 than in clusters 1 and 3 (figure 5A, D). Additionally, in the PROFILE cohort, participants in cluster 2 had significantly longer median survival (4·74 years [IQR 3·96–5·73] than did those in cluster 1 (2·87 years [2·29–3·40]) and in cluster 3 (2·23 years [1·75–3·84]; p<0·0001; figure 4). The unadjusted median survival of participants in cluster 2 did not differ significantly from that of participants in cluster 1 or cluster 3 in the replication cohort (appendix p 9), but was similar to that of participants in cluster 2 in the PROFILE cohort when adjusted for baseline lung function (appendix p 10).

Participants in cluster 3 showed an initial decline in lung function with subsequent stabilisation, and this cluster was the second most common cluster in both cohorts (figure 3E, F; table 1). This cluster was associated with high mortality (figure 4; table 1), high FEV_1_ to FVC ratio (figure 5), and high concentrations of PRO-C28 (figure 5C). Similarly, high mortality was observed among participants in cluster 3 in the replication cohort (appendix p 10).

Cluster 4 represented the smallest group of patients in both cohorts and reflected stable lung function over 3 years (figures 3, 4). Participants were younger (table 1) and had low concentrations of SP-D, but a tendency for high concentrations of RE-C1M and the lowest FEV_1_ to FVC ratio (figure 5). Cluster 1 had the highest number of ever smokers in the PROFILE cohort, although this was not significantly associated (table 1). This cluster had the longest median survival of the PROFILE cohort (5·56 years [95% CI 5·18–6·62]), differing significantly from that of cluster 1 (p<0·0001), cluster 2 (p=0·03), and cluster 3 (p<0·0001; figure 4). Although cluster architecture was similar between the PROFILE cohort and the replication cohort, mortality was higher in the replication cohort, even after adjusting for baseline FVC (appendix p 10).

The genetic analysis of common variants of idiopathic pulmonary fibrosis between the four clusters showed some nominal associations, but nothing of significance. Furthermore, no cluster was found to be associated with frequency of the at-risk MUC-5B minor allele (table 1; appendix p 15).

## Discussion

This study used machine learning methods to analyse lung function trajectories in two cohorts of patients with idiopathic pulmonary fibrosis. Using a random forest MCMC approach, we overcame the challenges associated with missing data and low statistical power in simple interpolation methods, such as last observation carried forward and simple linear regression.^4,6,8,12,13^ Conventional linear regression was acceptable for imputing data in the first year, similar to previous studies, including 12-month daily home spirometry studies.^22,23^ However, in these studies, a degree of heterogeneity exists that is not observed by regression to the mean, even in home spirometry studies that are often limited to short durations.^22,23^

We applied a model-based cluster analysis that, following a series of internal sensitivity and validity analyses, showed four discrete clusters of lung function trajectory. These clusters were associated with distinct anthropometric features with important implications for clinical management and future clinical trial design.

Cluster analysis in interstitial lung disease is an emerging concept. At least three studies have performed such analyses using registry cohorts, integrating various clinical features (including comorbidities) in an attempt to identify distinct phenotypes.^24–26^ However, these studies did not seek to identify discrete patterns of disease behaviour in patients with idiopathic pulmonary fibrosis. Our analyses identified four distinct FVC trajectories, which challenge the current understanding of the natural history of idiopathic pulmonary fibrosis.^3,6,7,10–12,27^ Patients in clusters 1 and 3 showed disease trajectories that followed the expected decline in lung function over the first year, and this continued throughout the duration of illness for patients in cluster 1, but stabilised for patients in cluster 3. Patients in clusters 1 and 3 were, unsurprisingly, more likely to have data missing due to death. More surprisingly, a third of patients in the overall cohort followed an alternative trajectory (ie, clusters 2 and 4) and showed either improved or stable lung function in the first year followed by a conventional trajectory (cluster 2), or remained stable throughout the duration of the study (cluster 4). Clusters 2 and 4 were associated with a better prognosis in patients with incident idiopathic pulmonary fibrosis than were clusters 1 and 3. Similar findings were found in a post-hoc analysis of the INPULSIS studies, which investigated the efficacy and safety of nintedanib added to pirfenidone in patients with idiopathic pulmonary fibrosis. However, this analysis was performed without imputation, which might underestimate the effect in patients receiving placebo and lead to immortal time bias in favour of therapy, thus reinforcing the need to undertake imputation in such analyses.^4^

The reasons behind the improvement in lung function among participants in cluster 2 are unclear, but there are several possible explanations. These patients might have had acute, or infective, exacerbations at enrolment into both studies that improved before the typically observed deterioration in lung function occurred.^28^ Nevertheless, this potential reason is unlikely given that it would require over 20% of patients with idiopathic pulmonary fibrosis to have an acute or infective exacerbation within a 6-month period of enrolment into both studies. Although such exacerbations are common, most estimates of incidence of acute exacerbations are lower than 20% within 1 year and are associated with poor prognosis.^3,28^ Another explanation could be that cluster 2 included patients with concomitant chronic obstructive pulmonary disease who showed labile results on spirometry.^29^ Compared with the other three clusters, cluster 2 contained more ever smokers and the FEV_1_ to FVC ratio was lower; however, this cluster was not associated with a lower diffusion capacity for carbon monoxide, suggesting that these patients did not have substantial emphysema, the form of chronic obstructive pulmonary disease most commonly associated with idiopathic pulmonary fibrosis.^30^ The disease trajectory in cluster 2 might reflect response to antifibrotic or immunosuppressive therapy, although patients in both studies were not receiving antifibrotic therapy at the time of recruitment, and treatment in idiopathic pulmonary fibrosis slows disease progression, rather than improves lung function.^5,6^ Furthermore, it is possible that individual variation in FVC values might have resulted in unusual patterns of lung function following cluster analysis; however, this is unlikely given the large number of patients in cluster 2 and that the nature and size of the cluster were replicated in the replication cohort. Although the reasons for the observed increase in FVC over the first year in cluster 2 are yet to be elucidated, it is important to recognise its occurrence in a substantial proportion of patients with idiopathic pulmonary fibrosis. Failure to recognise this occurrence could mislead interpretation of clinical response if unequal randomisation occurs in trials. Importantly, in the CAPACITY1 study and the CAPACITY2 study,^6^ the groups receiving placebo showed a different disease trajectory to those receiving pirfenidone, which ultimately delayed the regulatory approvals and introduction of this treatment into clinical practice. It is possible that this finding might have been due to the inclusion of patients showing cluster 2 or 4 trajectories in the placebo groups, who, combined, made up 40% of the patient cohort with idiopathic pulmonary fibrosis in both the PROFILE cohort and the replication cohort.

Identifying patients who are likely to show the disease trajectories of clusters 2 and 4 could have practical implications for clinical management. The notion of a therapeutic trial would be misleading for patients in cluster 2, who are likely to show an improvement in FVC despite, rather than because of, therapy. Furthermore, the risk–benefit ratio of any given therapy might be altered among patients in cluster 4, particularly those recently diagnosed with an FVC of more than 80%. However, further studies to prospectively test the predictive power of such models are needed.

There are various strengths to the approach used in this study. We used several validity and sensitivity analyses to identify optimal imputation methods over both the short and long term. Importantly, when trying to define natural history, we were able to analyse data from a prospective cohort of largely untreated patients to generate the imputation models and to identify the clusters of lung function trajectory. Additionally, we were able to replicate the clusters’ architecture in an external cohort of patients with idiopathic pulmonary fibrosis, and both cohorts shared some common anthropometric features.

However, our study also has several limitations. Due to the extent of missing data, there was only a small number of patients with idiopathic pulmonary fibrosis to effectively train the imputation algorithm, which might reduce the model’s ability to effectively identify further smaller clusters.^21^ Missing data are a challenge for all studies of idiopathic pulmonary fibrosis, both reducing statistical power and promoting survival bias in studies of individuals with idiopathic pulmonary fibrosis.^9,18^ We observed that a random forest-based imputation method had the lowest NRMSD, particularly at later timepoints 2 years or more from baseline, suggesting that machine learning approaches were most appropriate for these studies. However, we acknowledge that any imputation algorithm might not reflect the accurate decline in FVC, especially over longer periods of time. We also recognise that this approach might also introduce potential imputation biases, affecting cluster formation and subsequent interpretation of results. However, we believe that the advantages of data imputation with machine learning over standard interpolation models, as well as the extended sensitivity, validity, and replication analyses performed, substantially outweigh these limitations. Heterogeneity of individual lung function trajectories exists within clusters, which is unsurprising given the nature of lung function decline in patients with idiopathic pulmonary fibrosis.^5,11^ Nevertheless, this heterogeneity does not detract from strategies to stratify patients in clinical studies, given that, until now, all lung function trajectories were considered to be a uniform cluster. A further limitation to the current study is the absence of unadjusted replication of the relationship between cluster and mortality signal between the PROFILE cohort and the replication cohort. This difference might reflect the different nature of the two cohorts; PROFILE was a prospective cohort of patients with incident idiopathic pulmonary fibrosis, whereas the replication cohort was obtained from a registry cohort of patients with prevalent idiopathic pulmonary fibrosis and substantially less lung function at entry into the cohort. The small number of patients in cluster 4 of the replication cohort might have amplified the mortality signal. However, following adjustment for baseline FVC, clusters 1, 2, and 3 had similar survival in both cohorts.

This study identifies distinct trajectories of lung function in patients with idiopathic pulmonary fibrosis and has important implications for the development of clinical trials and clinical practice. Further improvement in collection of patient registry data and cluster methodology, as well as collaboration between research groups, will increase the accuracy of imputation and granularity of cluster analysis, thus facilitating further understanding of unique clusters of patients with pulmonary fibrosis, including those with pulmonary fibrosis of known cause. Development of these approaches could help to treat each patient with the correct treatment at the correct time.

## Supplementary Material

Supplement

## Figures and Tables

**Table 1:** Clinical characteristics per cluster in the PROFILE cohort

	Total	Cluster 1	Cluster 2	Cluster 3	Cluster 4

Number of patients	415 (100%)	140 (34%)	100 (24%)	113 (27%)	62 (15%)
Age, years	70·6 (7·9)	69·7 (7·6)	71·5 (8·0)	71·7 (7·7)	69·0 (8·3)
Sex					
Male	321	109 (78%)	82 (82%)	82 (73%)	48 (77%)
Female	94	31 (22%)	18 (18%)	31 (27%)	14 (23%)
Never smoker	117 (28%)	46 (33%)	24 (24%)	33 (29%)	14 (23%)
Ethnicity					
European descent	398 (96%)	134 (96%)	95 (95%)	110 (98%)	59 (95%)
Other	17 (4%)	6 (4%)	5 (5%)	3 (2%)	3 (5%)
Never used immunosuppressive medication	407 (98%)	137 (98%)	100 (100%)	111 (98%)	59 (95%)
Never used antifibrotic medication	303 (73%)	96 (70%)	70 (70%)	87 (77%)	50 (81%)
Completed all visits	82 (20%)	23 (16%)	24 (24%)	18 (16%)	17 (27%)
Missed visit at random	160 (39%)	44 (31%)	49 (49%)	32 (28%)	35 (56%)
Missed visit due to death	173 (42%)	73 (52%)	27 (27%)	63 (56%)	10 (16%)
Baseline percentile-predicted FVC	80·11 (19·23)	79·41 (19·80)	80·73 (18·69)	78·90 (18·92)	82·94 (19·49)
Baseline percentile-predicted diffusion capacity for carbon monoxide	46·03 (14·98)	43·24 (12·85)	48·66 (16·30)	44·42 (14·26)	51·33 (16·93)
Patients analysed due to missing data	380 (92%)	130 (93%)	89 (89%)	105 (93%)	56 (90%)
Baseline percentile-predicted FEVj	82·70 (18·1)	83·57 (19·1)	83·4 (17·9)	81·1 (17·6)	82·6 (17·1)
Patients analysed due to missing data	413 (100%)	138 (99%)	100 (100%)	113 (100%)	62 (100%)
Frequency of MUC-5B allele	34·67%	36·80%	36·56%	31·37%	35·85%
Patients analysed due to missing data	378 (91%)	125 (89%)	93 (93%)	102 (90%)	58 (94%)
<5-year survival	149 (36%)	32 (23%)	47(47%)	31 (27%)	39 (63%)
Survival, years	4·18 (3·87–4·37)	2·87 (2·29–3·40)	4·74 (3·96–5·73)	2·23 (1·75–3·84)	5·65 (5·18–6·62)

Data are n (%), mean (SD), or median (IQR). FVC=forced vital capacity. FEV_1_=forced expiratory volume in 1 s.

**Table 2:** Clinical characteristics per cluster in the Chicago cohort

	Total	Cluster 1	Cluster 2	Cluster 3	Cluster 4

Number of patients	180 (100%)	74 (41%)	38 (21%)	42 (23%)	26 (14%)
Age, years	68·4 (8·1)	67·8 (8·7)	69·5 (6·5)	69·1 (8·0)	67·6 (8·6)
Sex					
Male	141 (78%)	60 (81%)	31 (82%)	32 (76%)	18 (69%)
Female	39 (22%)	14 (19%)	7 (18%)	10 (24%)	8 (31%)
Never smoker	58 (32%)	28 (38%)	10 (26%)	11 (26%)	9 (35%)
Patients analysed due to missing data (smokers)	4 (2%)	2 (3%)	1 (3%)	1 (2%)	0
Ethnicity					
European descent	158 (88%)	69 (93%)	31 (82%)	39 (93%)	19 (73%)
Other	22 (12%)	5 (7%)	7 (18%)	3 (7%)	7 (27%)
Completed all visits	15 (8%)	4 (5%)	4 (11%)	2 (5%)	5 (19%)
Missed visit at random	78 (43%)	32 (43%)	17 (45%)	22 (52%)	7 (27%)
Missed visit due to death	87 (48%)	38 (51%)	17 (45%)	22 (43%)	14 (54%)
Baseline percentile-predicted FVC	69·20 (16·26)	71·80 (10·66)	64·66 (12·36)	74·83 (20·87)	59·27 (20·27)
<5-year survival	44 (24%)	15 (20%)	9 (24%)	13 (31%)	7 (27%)
Survival, years	3·65 (3·29–4·02)	2·87 (2·49–3·89)	2·98 (2·14–4·87)	3·41 (2·45–4·40)	2·64 (1·59–5·00)

Data are n (%), mean (SD), or median (IQR). FVC=forced vital capacity.

## Data Availability

The PROFILE (anonymously processed) dataset used in this study can be made available on a reasonable request basis, which must include an appropriate protocol, analysis plan, and data exchange with institutional approvals in place before data transfer of any information. This request needs to be formally addressed to the head of the Margaret Turner Warwick for Centre for Fibrosing Lung Disease, RGJ (gisli.jenkins@imperial.ac.uk). External validation data sources will not be provided because these are withheld by owners. Patient personal information and correspondence will not be provided because these are withheld by the corresponding author’s organisation to preserve patient privacy. The code of the method is freely available.

## Research in context

### Evidence before this study

We searched PubMed without date or language restrictions from date of database inception to Dec 9, 2021, using the following search terms: “idiopathic pulmonary fibrosis”, “lung function”, and “clustering” or “imputation”. We identified three studies that had performed a cluster analysis in retrospective or registry collections to identify clusters of patients with interstitial lung disease on the basis of various clinical features, including lung function. No previous study had combined data-driven and self-organising algorithms to understand the nature of long-term lung function trajectories in patients with idiopathic pulmonary fibrosis. Among studies that had imputed missing data on lung function, these data were often attributable to a linear change or population average, and no studies had assessed several methods for imputing missing data. No previous study used unsupervised self-organising maps to model and interpret long-term lung function to evaluate the presence of distinct lung function trajectories and how they associate with clinical outcomes.

### Added value of this study

To our knowledge, this is the first study to identify and validate lung function trajectories with a two-stage machine learning approach, including both supervised and unsupervised approaches, in a long-term prospective observational cohort of patients with incident idiopathic pulmonary fibrosis. Using a Markov Chain Monte-Carlo simulation approach, we were able to overcome the challenges associated with low statistical power due to missing data, often in cases where disease severity was a barrier to lung function testing. We performed an extensive series of internal sensitivity and validity analyses, as well as external replication, to provide robust conclusions. Our analyses showed that a model-based cluster analysis was able to find four discrete trajectories of longitudinal lung function in patients with idiopathic pulmonary fibrosis. These clusters were associated with distinct clinical and biochemical features that might have important implications for clinical management. Our machine learning analysis showed that two-thirds of patients followed a typically observed disease trajectory comprising a steady initial decline in lung function; however, a third of patients showed alternative trajectories with either improved or stable lung function overtime, complicating the interpretation of widely used endpoints in patients with idiopathic pulmonary fibrosis. Importantly, these clusters were associated with an improved prognosis in the cohort of treatment-naive patients with pulmonary fibrosis.

### Implications of all the available evidence

Our findings on the different lung function trajectories in patients with idiopathic pulmonary fibrosis could have major implications for research and patient care. Our imputation models provide valuable comparisons that can support evaluation of endpoints from data with non-random missingness. Stratification of patients by lung function cluster would support the design of clinical trials and effective randomisation to support the assessment of treatment-related effects and to minimise confounding of natural disease trajectories. Similarly, understanding the natural history of lung function in treatment-naive patients might help to inform their prognosis in the medium and long term on the basis of short-term changes in lung function.

## References

[1] Raghu G, Rochwerg B, Zhang Y, An official ATS/ERS/JRS/ALAT clinical practice guideline: treatment of idiopathic pulmonary fibrosis. An update of the 2011 clinical practice guideline. Am J Respir Crit Care Med 2015; 192: e3–19. 26177183 10.1164/rccm.201506-1063ST

[2] Nathan SD, Shlobin OA, Weir N, Long-term course and prognosis of idiopathic pulmonary fibrosis in the new millennium. Chest 2011; 140: 221–29. 21729893 10.1378/chest.10-2572

[3] Reichmann WM, Yu YF, Macaulay D, Wu EQ, Nathan SD. Change in forced vital capacity and associated subsequent outcomes in patients with newly diagnosed idiopathic pulmonary fibrosis. BMC Pulm Med 2015; 15: 167. 26714746 10.1186/s12890-015-0161-5 PMC4696269

[4] Flaherty KR, Fell CD, Huggins JT, Safety of nintedanib added to pirfenidone treatment for idiopathic pulmonary fibrosis. Eur Respir J 2018; 52: 1800230. 29946005 10.1183/13993003.00230-2018 PMC6092682

[5] Nathan SD, Albera C, Bradford WZ, Effect of continued treatment with pirfenidone following clinically meaningful declines in forced vital capacity: analysis of data from three phase 3 trials in patients with idiopathic pulmonary fibrosis. Thorax 2016; 71: 429–35. 26968970 10.1136/thoraxjnl-2015-207011 PMC4862066

[6] Noble PW, Albera C, Bradford WZ, Pirfenidone in patients with idiopathic pulmonary fibrosis (CAPACITY): two randomised trials. Lancet 2011; 377: 1760–69. 21571362 10.1016/S0140-6736(11)60405-4

[7] Richeldi L, du Bois RM, Raghu G, Efficacy and safety of nintedanib in idiopathic pulmonary fibrosis. N Engl J Med 2014; 370: 2071–82. 24836310 10.1056/NEJMoa1402584

[8] Lederer DJ, Bradford WZ, Fagan EA, Sensitivity analyses of the change in FVC in a phase 3 trial of pirfenidone for idiopathic pulmonary fibrosis. Chest 2015; 148: 196–201. 25856121 10.1378/chest.14-2817 PMC4493875

[9] Thabut G, Crestani B, Porcher R, Richeldi L. Missing data in IPF trials: do not let methodological issues undermine a major therapeutic breakthrough. Eur Respir J 2015; 46: 607–14. 26324692 10.1183/13993003.02204-2014

[10] Salisbury ML, Xia M, Zhou Y, Idiopathic pulmonary fibrosis: gender-age-physiology index stage for predicting future lung function decline. Chest 2016; 149: 491–98. 26425858 10.1378/chest.15-0530 PMC4944785

[11] Santermans E, Ford P, Kreuter M, Modelling forced vital capacity in idiopathic pulmonary fibrosis: optimising trial design. Adv Ther 2019; 36: 3059–70. 31565781 10.1007/s12325-019-01093-3 PMC6822798

[12] Behr J, Prasse A, Kreuter M, Pirfenidone in patients with progressive fibrotic interstitial lung diseases other than idiopathic pulmonary fibrosis (RELIEF): a double-blind, randomised, placebo-controlled, phase 2b trial. Lancet Respir Med 2021; 9: 476–86. 33798455 10.1016/S2213-2600(20)30554-3

[13] King TE Jr, Bradford WZ, Castro-Bernardini S, A phase 3 trial of pirfenidone in patients with idiopathic pulmonary fibrosis. N Engl J Med 2014; 370: 2083–92. 24836312 10.1056/NEJMoa1402582

[14] Beretta L, Santaniello A. Nearest neighbor imputation algorithms: a critical evaluation. BMC Med Inform Decis Mak 2016; 16 (suppl 3): 74. 27454392 10.1186/s12911-016-0318-z PMC4959387

[15] Kokla M, Virtanen J, Kolehmainen M, Paananen J, Hanhineva K. Random forest-based imputation outperforms other methods for imputing LC-MS metabolomics data: a comparative study. BMC Bioinformatics 2019; 20: 492. 31601178 10.1186/s12859-019-3110-0 PMC6788053

[16] Breiman L Random forests. Mach Learn 2001; 45: 5–32.

[17] Box GEP, Jenkins GM, Reinsel GC, Ljung GM. Time series analysis: forecasting and control, 5th edn. Hoboken, NJ: John Wiley and Sons, 2015.

[18] Buuren S Flexible imputation of missing data, 1st edn. New York, NY: Chapman and Hall, 2012.

[19] Jenkins RG, Simpson JK, Saini G, Longitudinal change in collagen degradation biomarkers in idiopathic pulmonary fibrosis: an analysis from the prospective, multicentre PROFILE study. Lancet Respir Med 2015; 3: 462–72. 25770676 10.1016/S2213-2600(15)00048-X

[20] Noth I, Zhang Y, Ma SF, Genetic variants associated with idiopathic pulmonary fibrosis susceptibility and mortality: a genome-wide association study. Lancet Respir Med 2013; 1: 309–17. 24429156 10.1016/S2213-2600(13)70045-6 PMC3894577

[21] Vesanto J, Alhoniemi E. Clustering of the self-organizing map. IEEE Trans Neural Netw 2000; 11: 586–600. 18249787 10.1109/72.846731

[22] Russell AM, Adamali H, Molyneaux PL, Daily home spirometry: an effective tool for detecting progression in idiopathic pulmonary fibrosis. Am J Respir Crit Care Med 2016; 194: 989–97. 27089018 10.1164/rccm.201511-2152OC PMC5067818

[23] Maher TM, Corte TJ, Fischer A, Pirfenidone in patients with unclassifiable progressive fibrosing interstitial lung disease: a double-blind, randomised, placebo-controlled, phase 2 trial. Lancet Respir Med 2020; 8: 147–57. 31578169 10.1016/S2213-2600(19)30341-8

[24] Aoshima Y, Karayama M, Horiike Y, Cluster analysis-based clinical phenotypes of idiopathic interstitial pneumonias: associations with acute exacerbation and overall survival. BMC Pulm Med 2021; 21: 63. 33618682 10.1186/s12890-021-01428-3 PMC7898746

[25] Gao J, Kalafatis D, Carlson L, Baseline characteristics and survival of patients of idiopathic pulmonary fibrosis: a longitudinal analysis of the Swedish IPF Registry. Respir Res 2021; 22: 40. 33546682 10.1186/s12931-021-01634-x PMC7866760

[26] Wong AW, Lee TY, Johannson KA, A cluster-based analysis evaluating the impact of comorbidities in fibrotic interstitial lung disease. Respir Res 2020; 21: 322. 33287805 10.1186/s12931-020-01579-7 PMC7720501

[27] Schmidt SL, Nambiar AM, Tayob N, Pulmonary function measures predict mortality differently in IPF versus combined pulmonary fibrosis and emphysema. Eur Respir J 2011; 38: 176–83. 21148225 10.1183/09031936.00114010 PMC4084829

[28] Collard HR, Ryerson CJ, Corte TJ, Acute exacerbation of idiopathic pulmonary fibrosis. An international working group report. Am J Respir Crit Care Med 2016; 194: 265–75. 27299520 10.1164/rccm.201604-0801CI

[29] Cordier JF, Cottin V. Neglected evidence in idiopathic pulmonary fibrosis: from history to earlier diagnosis. Eur Respir J 2013; 42: 916–23. 23598958 10.1183/09031936.00027913

[30] Kwiatkowska S IPF and CPFE - the two different entities or two different presentations of the same disease? Adv Respir Med 2018; 86: 23–26. 29286173 10.5603/ARM.a2017.0049

