# Supplementary material for: Forced vital capacity trajectories in patients with idiopathic pulmonary fibrosis: a secondary analysis of a multicentre, prospective, observational cohort
Source: Lancet Digit Health. Author manuscript; Available in PMC 2025 Nov 25. (PMC12646456; doi:10.1016/S2589-7500(22)00173-X)
Supplement: Supplement [file NIHMS1937503-supplement-Supplement.pdf]

THE LANCET
Digital Health

Supplementary appendix

This appendix formed part of the original submission and has been peer reviewed. We post it as supplied by the authors.

Supplement to: Fainberg HP, Oldham JM, Molyneau PL, et al. Forced vital capacity trajectories in patients with idiopathic pulmonary fibrosis: a secondary analysis of a multicentre, prospective, observational cohort. *Lancet Digit Health* 2022; published online Nov 1. https://doi.org/10.1016/ S2589-7500(22)00173-X.

<u>Supplementary data section</u>: Analysis of forced vital capacity (FVC) trajectories in Idiopathic Pulmonary Fibrosis (IPF) identifies four distinct clusters of disease behaviour.

Contents

Section 1	Methods	2
	Study Population	2
	Serum Biomarkers	2
	K Means Clustering	3
	Analysis of genetic common variants associated with IPF susceptibility	3
Section 2	Figures and tables	4
	Supplemental Figure 1	4
	Supplemental Figure 2	5
	Supplemental Figure 3	6
	Supplemental Figure 4	7
	Supplemental Figure 5	8
	Supplemental Figure 6	9
	Supplemental Figure 7	10
	Supplemental Figure 8	11
	Supplemental Figure 9	12
	Supplemental Figure 10	13
	Supplemental Table 1	14
	Supplemental Table 2	15
	Supplemental Table 3	16
Section 3	Legends	18
	Figure Legends	18
	Table Legends	22
Section 4	References	23

Section 1: Methods

Study population

The PROFILE study is a multicentre, prospective, observational, longitudinal cohort study of incident IPF described previously (1). Patient recruitment started in 2010, and the final spirometric visit was in 2018. PROFILE consisted of 581 participants enrolled through co-ordinating centre in Central England (NCT01134822) and the Royal Brompton Hospital (NCT01110694).

Participants who completed at least the baseline and three-month or six-month spirometry visits were selected for further analysis. Most patients did not receive anti-fibrotic therapy as visits largely occurred prior to approval of these treatments within National Health Service. Mortality data were censored on May 01, 2021.

Independent replication included a case-control study previously described by Noth et al (2). In brief, the Chicago Consortium recruited 455 participants, including 180 with spirometry records meeting the a-priori requirements for further analysis. This independent longitudinal spirometric data was harmonised to the PROFILE study visit time frame to ensure comparability of similar measurements **(Suppl. Table 2).**

All studies diagnosed cases using American Thoracic and European Respiratory Societies guidelines and had appropriate institutional review board and ethics approval

Serum Biomarkers

Serum biomarkers were measured from samples prospectively collected at baseline and analysed as previously described (1). a) ELISA-based assays of extracellular matrix derived neoepitopes including: C3M (type-III collagen-degraded by MMP-9), C6M (type-VI collagen-degraded by MMP-2), PRO-C3 (N-terminal pro-peptide of type-III collagen), PRO-C6 (C5 domain of type-VI collagen - endotrophin), RE-C1M (type-I collagen-degraded by MMP-2/9/13) and PRO-C28 (C-terminal fragment of type-XXVIII

collagen); b) Capture ELISA-panel which included MMP-7, serum surfactant protein-D (SPD) and cytokeratin fragment-19 (CYFRA211).

K-means clustering

K-means clustering was also implemented in R. First, the input data was normalised using the same pre-processing step as in SOM algorithm. This cluster algorithm was applied in an unsupervised manner as the SOM method (3).

Analysis of genetic common variants associated with IPF susceptibility

We compared individuals in a single cluster with individuals in the other three clusters and tested whether there was a significant difference in allele frequency for 20 genetic variants that have been previously reported to be associated with IPF susceptibility (4). We used a logistic regression model, adjusting for recruitment centre and the first 10 genetic principal components. Nominal significance was set at 0.05 and the threshold for Bonferroni-corrected significance was < 6.25e-4.

Section 2:

Figures and tables

Suppl. fig 1

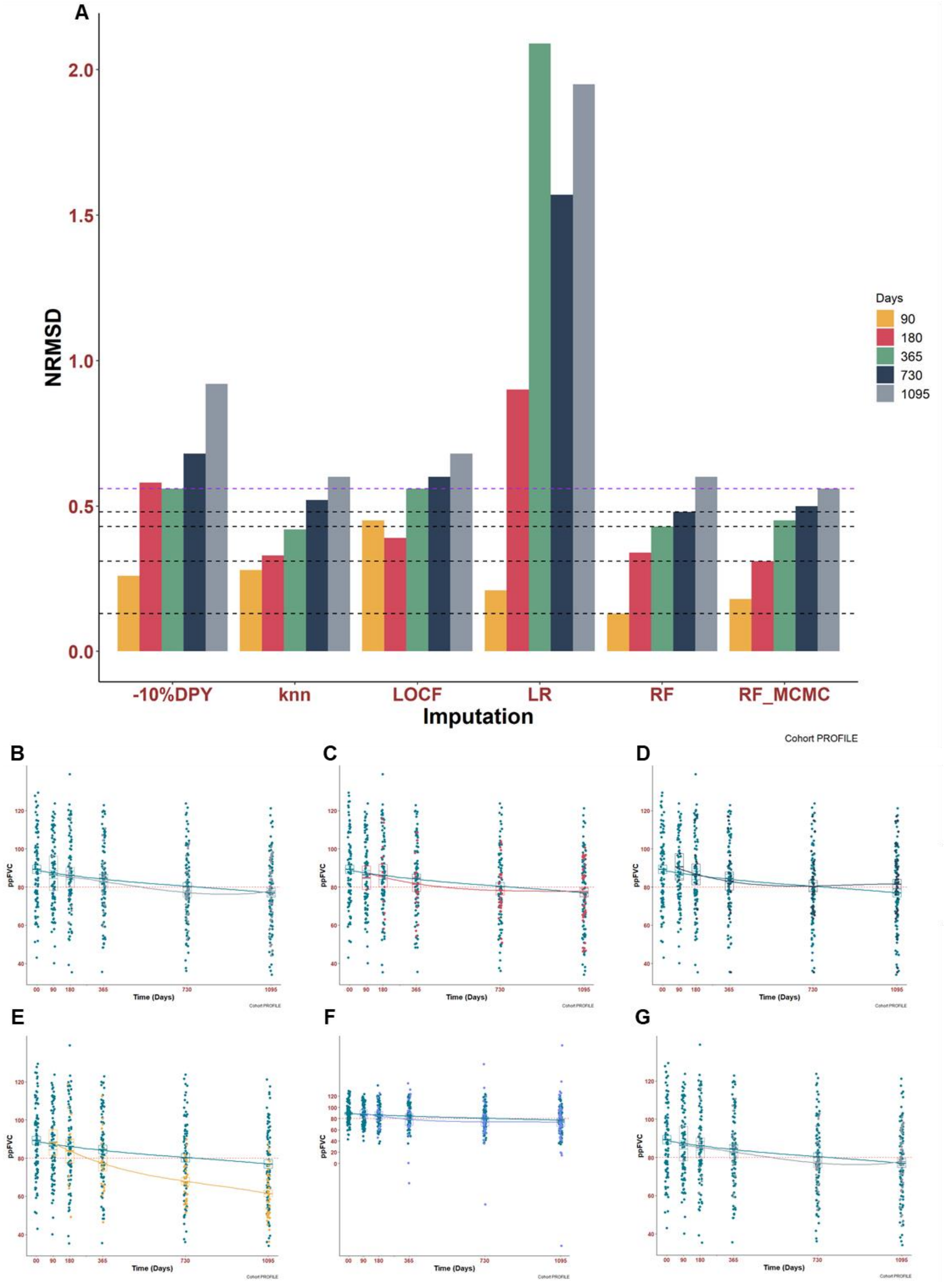

Suppl. fig 2

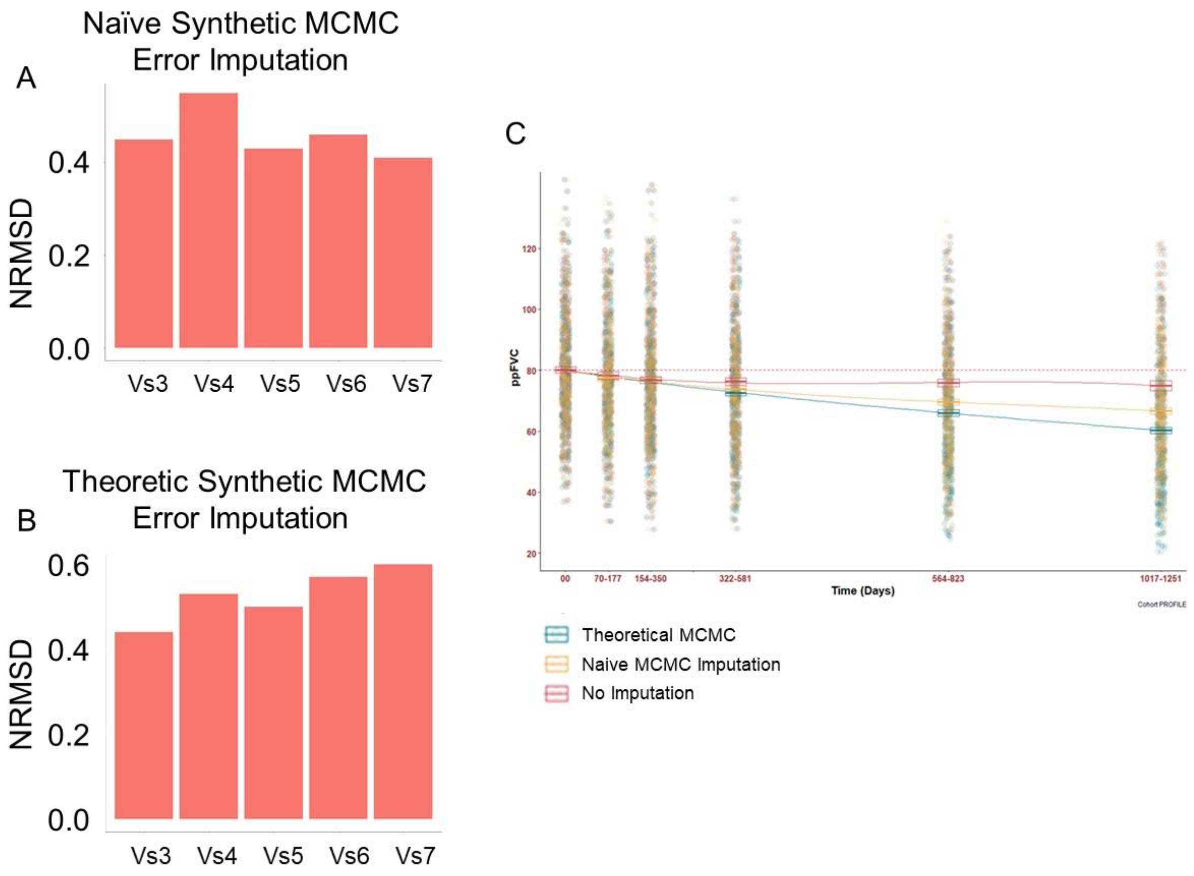
A
Naïve Synthetic MCMC
Error Imputation
NRMSD
0.0
0.2
0.4
Vs3
Vs4
Vs5
Vs6
Vs7
B
Theoretic Synthetic MCMC
Error Imputation
NRMSD
0.0
0.2
0.4
0.6
Vs3
Vs4
Vs5
Vs6
Vs7
C
ppFVC
20
40
60
80
100
120
00
70-177
154-350
322-581
564-823
1017-1251
Time (Days)
Cohort PROFILE
Theoretical MCMC
Naive MCMC Imputation
No Imputation

Suppl. fig 3

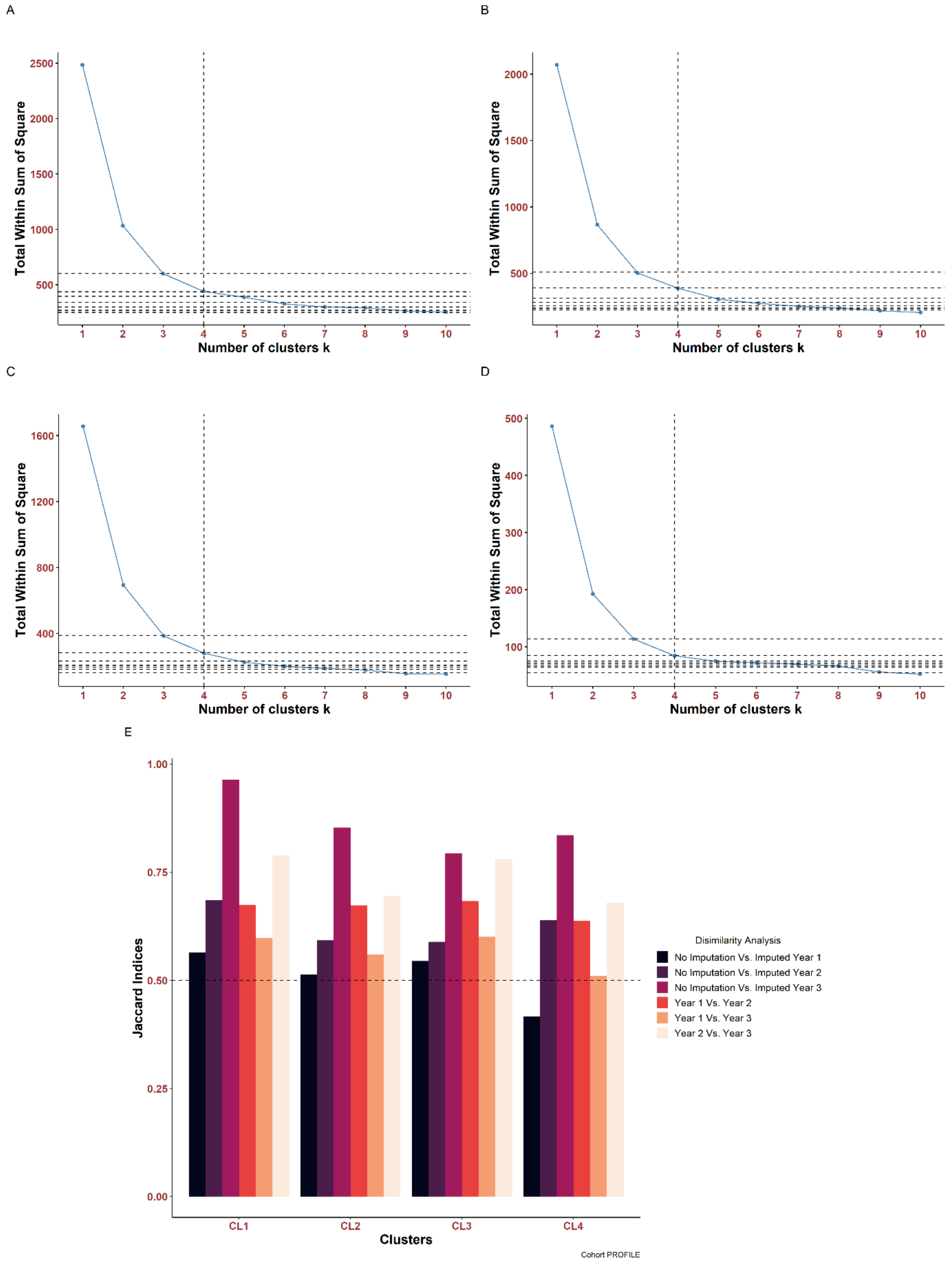
A
B
C
D
E
Total Within Sum of Square
Number of clusters k
2500
2000
1500
1000
500
1600
1200
800
400
300
200
100
1 2 3 4 5 6 7 8 9 10
Jaccard Indices
1.00
0.75
0.50
0.25
0.00
CL1
CL2
CL3
CL4
Clusters
Disimilarity Analysis
No Imputation Vs. Imputed Year 1
No Imputation Vs. Imputed Year 2
No Imputation Vs. Imputed Year 3
Year 1 Vs. Year 2
Year 1 Vs. Year 3
Year 2 Vs. Year 3
Cohort PROFILE

Suppl. fig 4

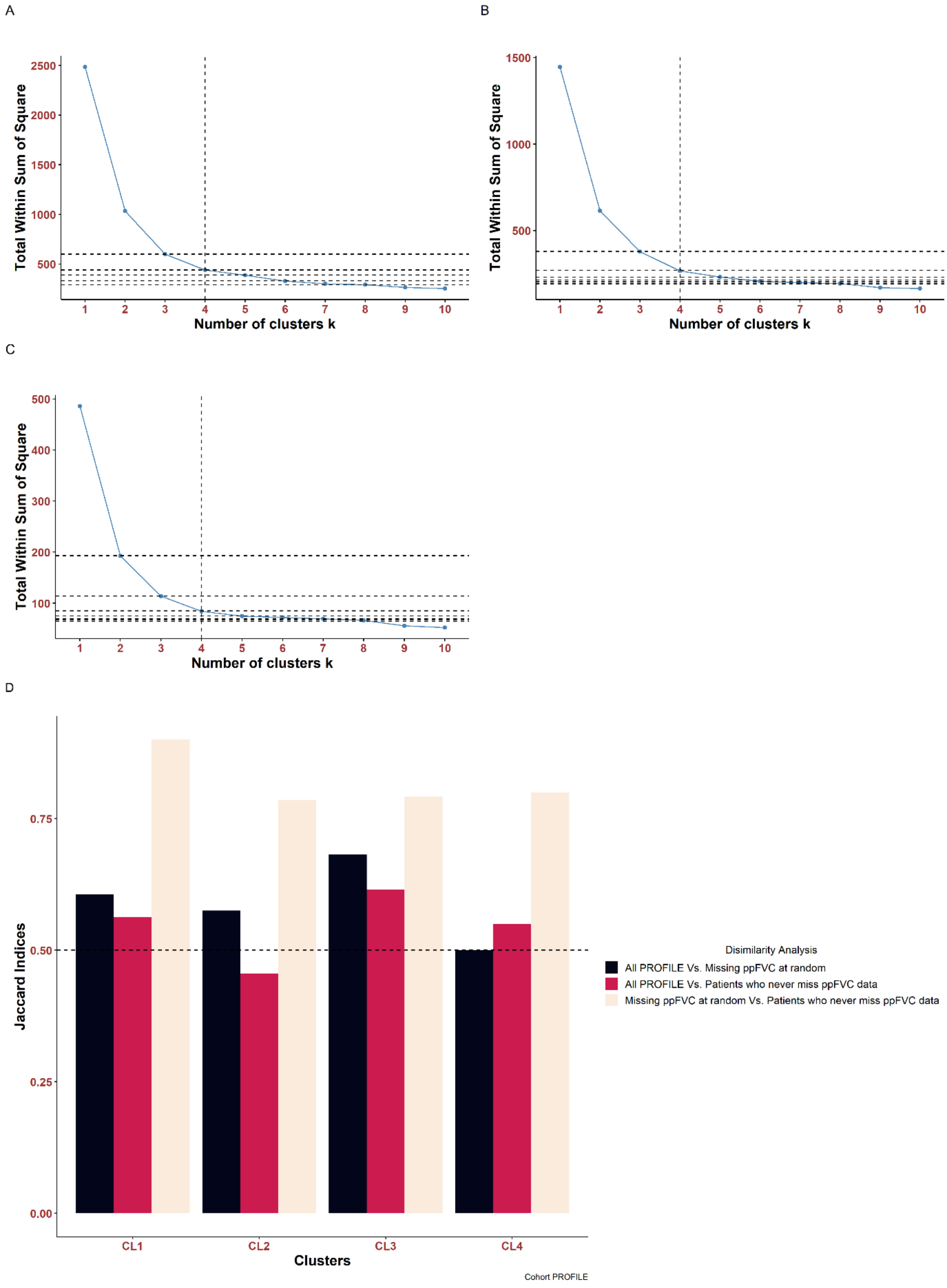

A
Total Within Sum of Square
2500
2000
1500
1000
500
1 2 3 4 5 6 7 8 9 10
Number of clusters k
B
Total Within Sum of Square
1500
1000
500
1 2 3 4 5 6 7 8 9 10
Number of clusters k
C
Total Within Sum of Square
500
400
300
200
100
1 2 3 4 5 6 7 8 9 10
Number of clusters k
D
Jaccard Indices
0.75
0.50
0.25
0.00
CL1
CL2
CL3
CL4
Clusters
Disimilarity Analysis
All PROFILE Vs. Missing ppFVC at random
All PROFILE Vs. Patients who never miss ppFVC data
Missing ppFVC at random Vs. Patients who never miss ppFVC data
Cohort PROFILE

Suppl. fig 5

A

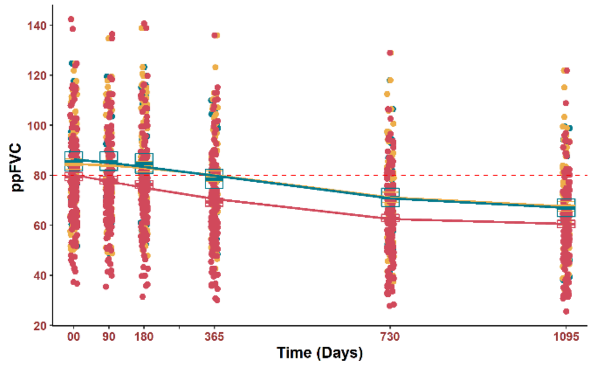

	Beta	95% CI	t.value	Sig
(Intercept)	76.04	[74.80 - 77.28]	120.356	***
Missing ppFVC data due to Death	-4.99	[-6.46 - -3.52]	-6.640	***
Missing ppFVC data at random	2.31	[0.71 - 3.90]	2.839	**
Missing ppFVC data due to Death:(ppFVC Adjusted)	-0.31	[-1.24 - -0.61]	-0.665	
Missing ppFVC data at random:(ppFVC Adjusted)	0.17	[-0.69 - 1.03]	0.386	

B

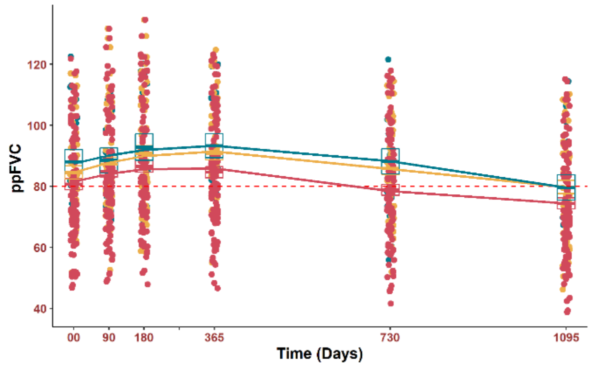

	Beta	95% CI	t.value	Sig
(Intercept)	85.56	[84.19 - 86.92]	122.856	***
Missing ppFVC data due to Death	-3.87	[-5.48 - -2.26]	-4.715	***
Missing ppFVC data at random	0.99	[-0.77 - -2.75]	1.099	
Missing ppFVC data due to Death:(ppFVC Adjusted)	-0.12	[-0.91- -0.67]	0.762	
Missing ppFVC data at random:(ppFVC Adjusted)	0.04	[-0.71- 0.80]	0.912	

C

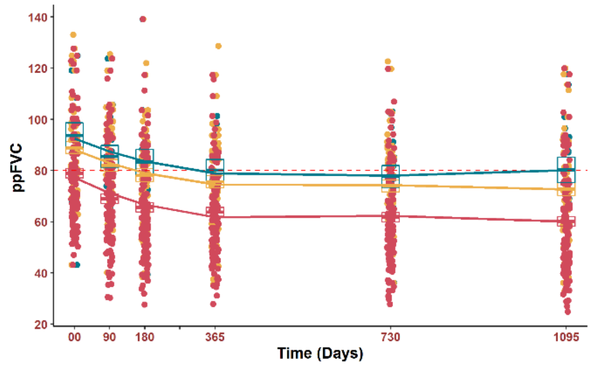

	Beta	95% CI	t.value	Sig
(Intercept)	76.19	[74.70 - 77.68]	100.180	***
Missing ppFVC data due to Death	-9.65	[-11- -7.92]	-10.958	***
Missing ppFVC data at random	2.40	[0.49 - 4.30]	2.466	*
Missing ppFVC data due to Death:(ppFVC Adjusted)	-0.38	[-1.33 - -0.58]	0.440	
Missing ppFVC data at random:(ppFVC Adjusted)	0.17	[-0.73 -1.08]	0.371	

D

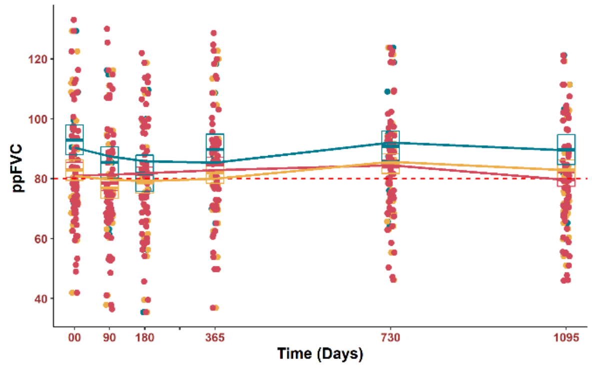

	Beta	95% CI	t.value	Sig
(Intercept)	83.90	[82.10 - 85.70]	91.608	***
Missing ppFVC data due to Death	-2.04	[-4.16 - 0.08]	-1.887	
Missing ppFVC data at random	-2.49	[-4.86 - 0.12]	-2.066	*
Missing ppFVC data due to Death:(ppFVC Adjusted)	-0.03	[-1.14 - 1.07]	-0.060	
Missing ppFVC data at random:(ppFVC Adjusted)	-0.04	[1.01 - 1.01]	-0.083	

Suppl. fig 6

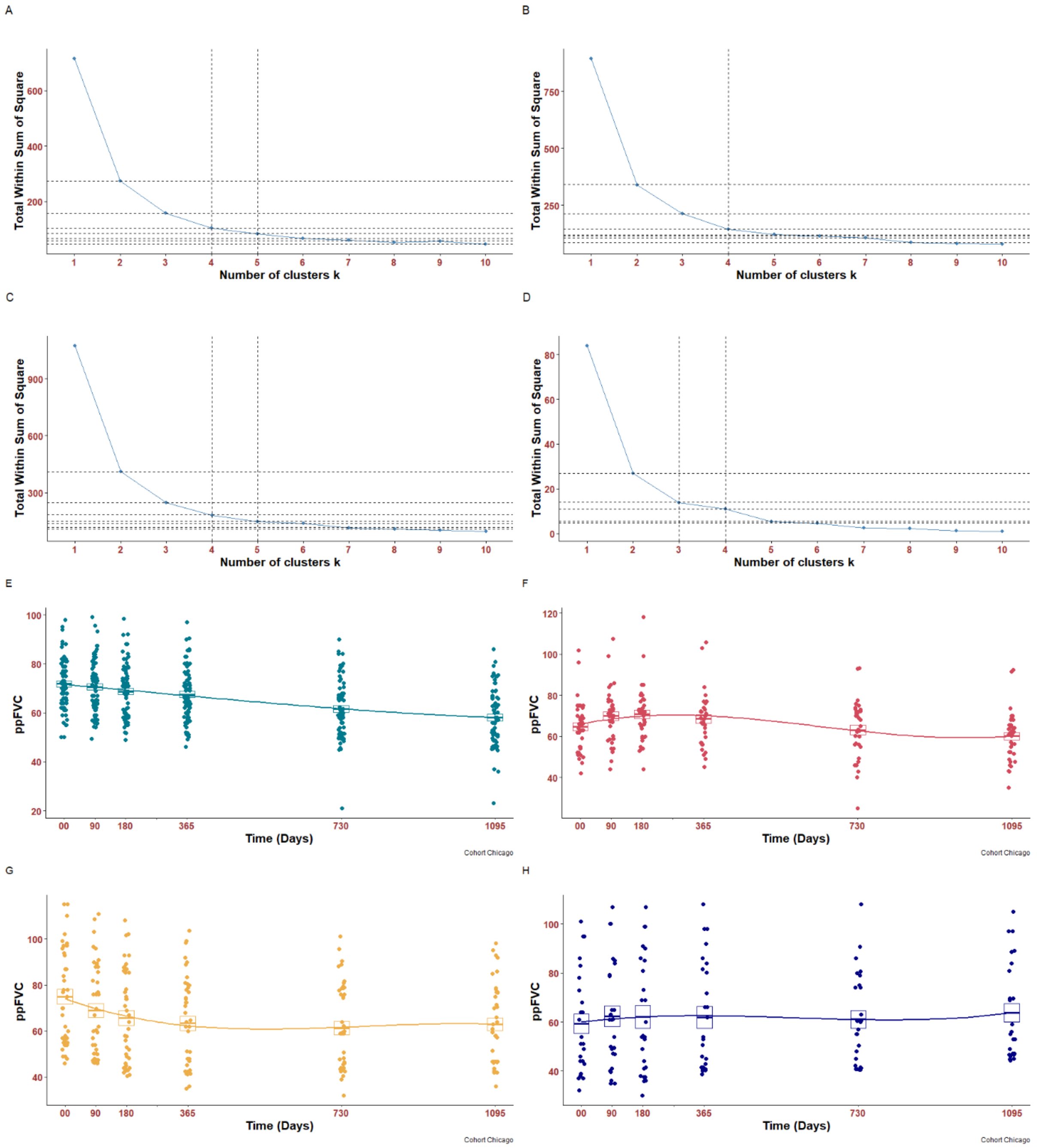
A
B
C
D
E
F
G
H
Total Within Sum of Square
Number of clusters k
ppFVC
Time (Days)
Cohort Chicago

Suppl. fig 7

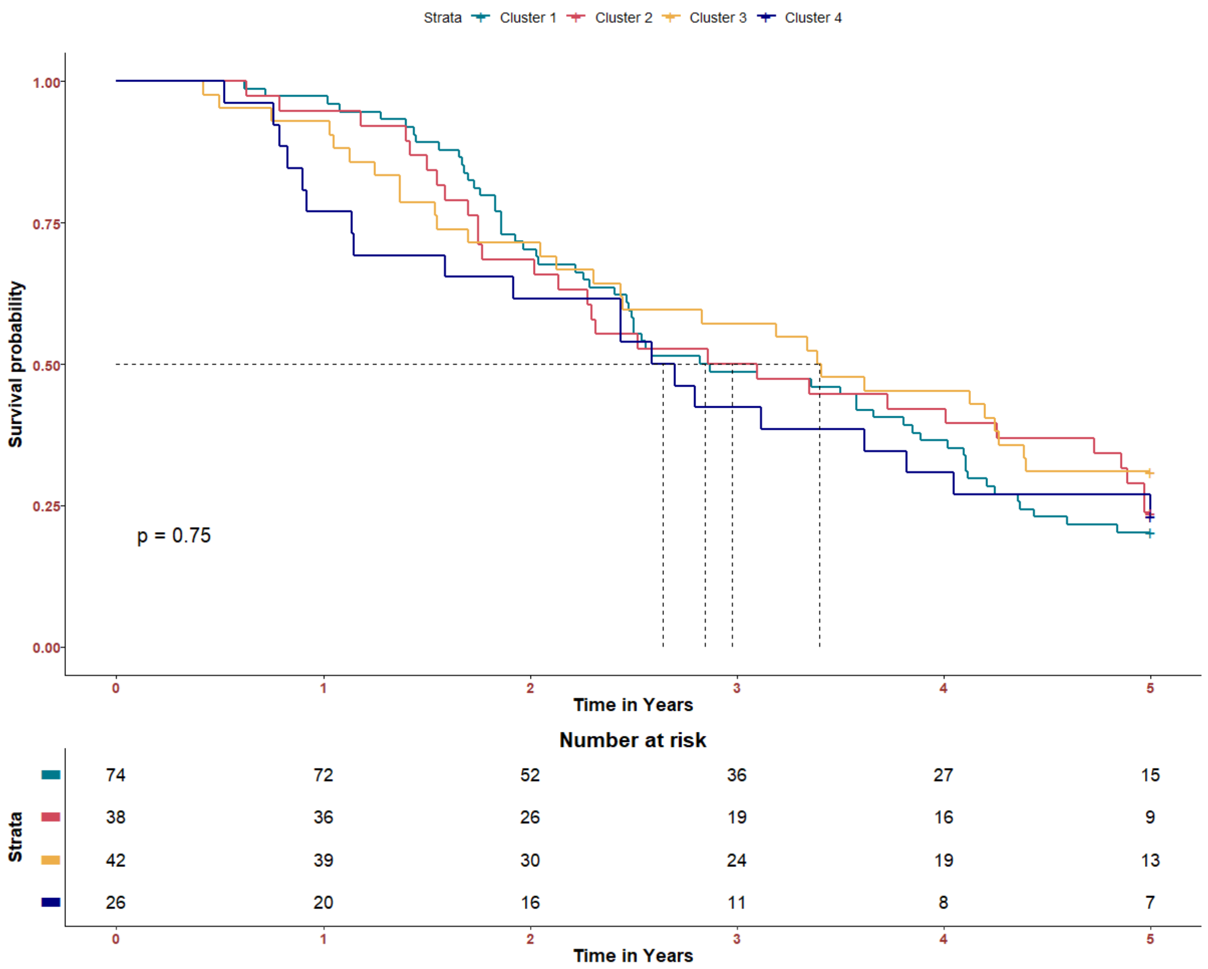
Strata Cluster 1 Cluster 2 Cluster 3 Cluster 4
Survival probability
1.00
0.75
0.50
0.25
0.00
p = 0.75
0 1 2 3 4 5
Time in Years
Number at risk
Strata
74 72 52 36 27 15
38 36 26 19 16 9
42 39 30 24 19 13
26 20 16 11 8 7
0 1 2 3 4 5
Time in Years

Suppl. fig 8

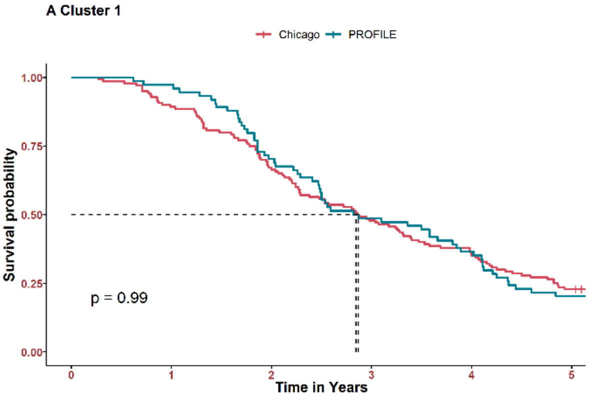

Study	OR	95% CI
PROFILE-HR	1.10	[0.64-1.89]
Replication-HR	1.54	[0.88-2.81]
PROFILE-HR (AdjustedBaseline FVC)	1.31	[0.75-2.26]
Replication-HR (Adjusted Baseline FVC)	1.53	[0.65-2.81]

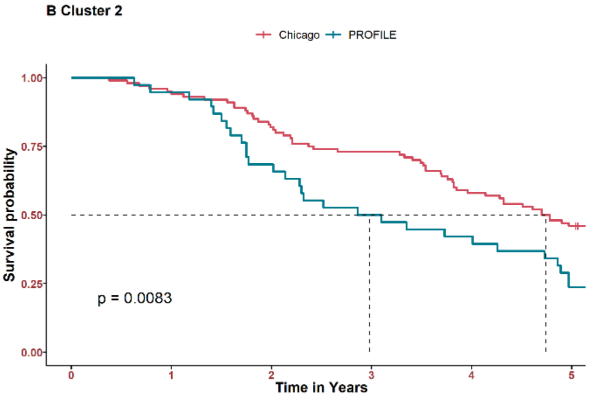

Study	OR	95% CI
PROFILE-HR	0.62	[0.35-1.12]
Replication-HR	2.33	[1.10-4.93]
PROFILE-HR (AdjustedBaseline FVC)	1.08	[0.57-1.96]
Replication-HR (Adjusted Baseline FVC)	1.15	[0.53-2.51]

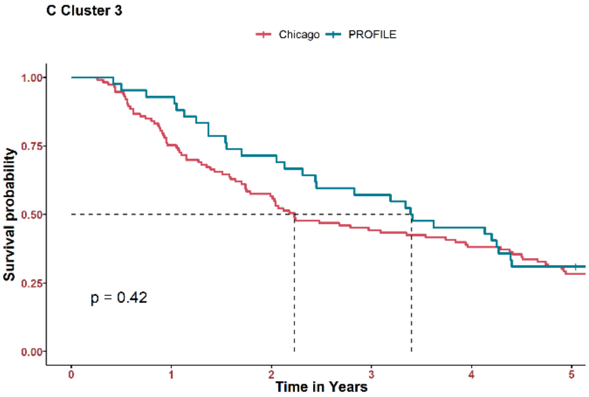

Study	OR	95% CI
PROFILE-HR	1.34	[0.75-2.41]
Replication-HR	0.90	[0.46-1.70]
PROFILE-HR (AdjustedBaseline FVC)	1.35	[0.90-2.96]
Replication-HR (Adjusted Baseline FVC)	0.95	[0.52-1.82]

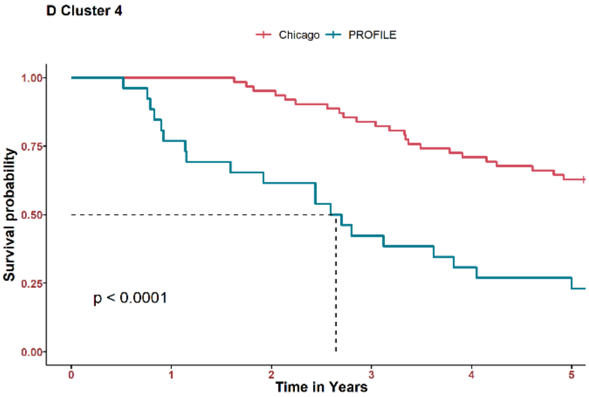

Study	OR	95% CI
PROFILE-HR	0.27	[0.14-0.51]
Replication-HR	3.09	[1.41-6.82]
PROFILE-HR (AdjustedBaseline FVC)	0.49	[0.24-1.01]
Replication-HR (Adjusted Baseline FVC)	1.81	[0.77-4.30]

Suppl. fig 9

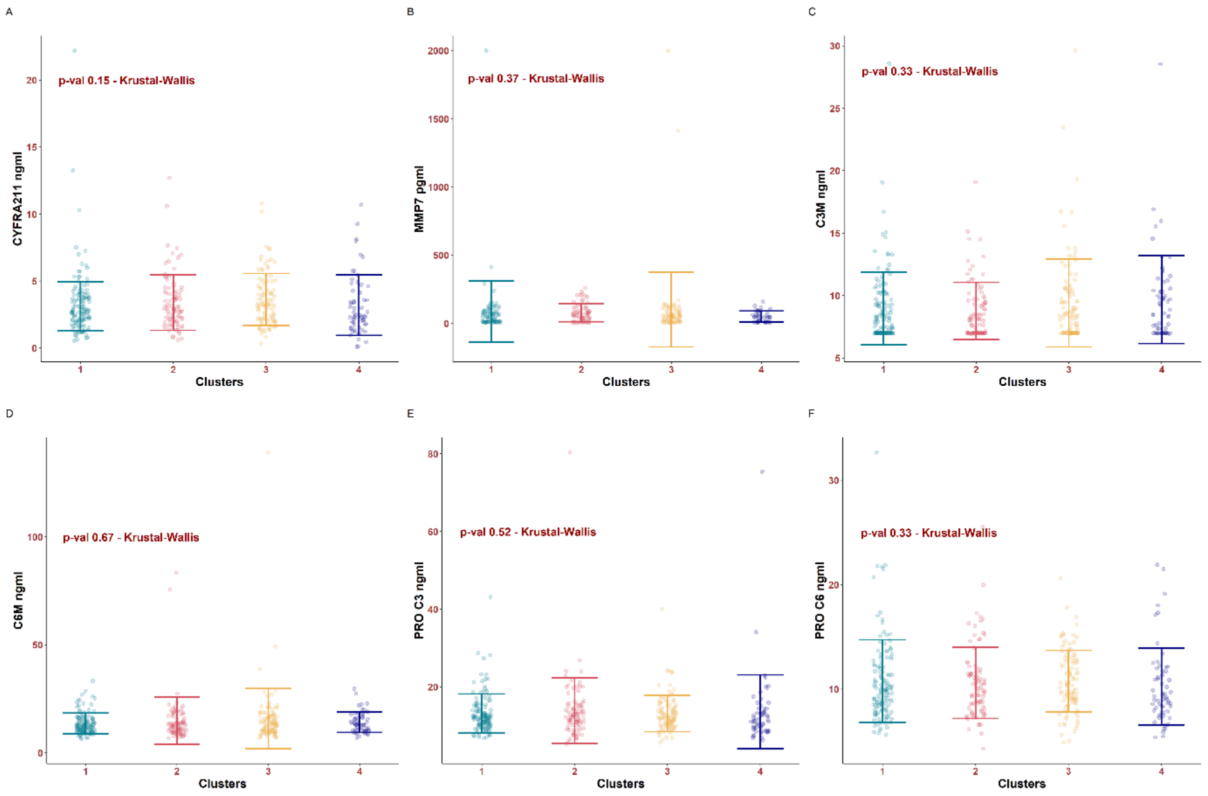
A
p-val 0.15 - Krustal-Wallis
CYFRA211 ngml
Clusters
B
p-val 0.37 - Krustal-Wallis
MMP7 pgml
Clusters
C
p-val 0.33 - Krustal-Wallis
C3M ngml
Clusters
D
p-val 0.67 - Krustal-Wallis
C6M ngml
Clusters
E
p-val 0.52 - Krustal-Wallis
PRO C3 ngml
Clusters
F
p-val 0.33 - Krustal-Wallis
PRO C6 ngml
Clusters

Suppl. fig 10

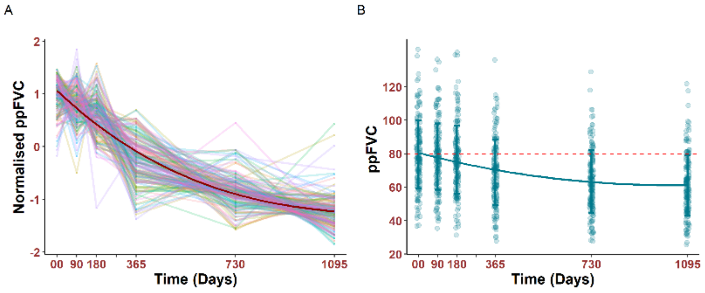

Cluster 1 - n=159				
**Days**	**ppFVC**	**sd**	**sem**	**95% CI**
1	79.68	20.20	1.61	76.51-82.86
90	78.31	19.80	1.58	75.2-81.42
180	76.43	20.48	1.63	73.21-79.65
365	68.90	19.64	1.56	65.81-71.98
730	63.52	18.68	1.49	60.59-66.45
1,095	61.01	18.15	1.44	58.15-63.86

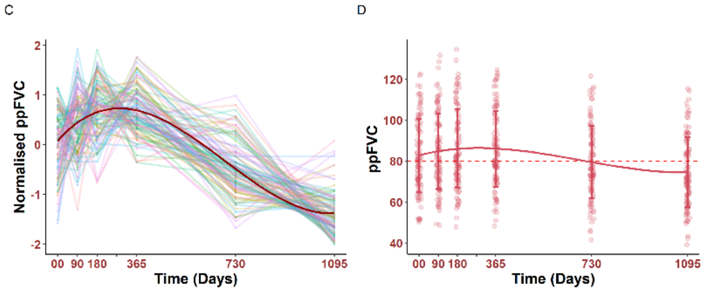

Cluster 2 - n=100				
**Days**	**ppFVC**	**sd**	**sem**	**95% CI**
1	82.75	17.85	1.79	79.19-86.31
90	84.78	18.45	1.85	81.1-88.46
180	86.24	19.04	1.91	82.44-90.04
365	85.96	18.62	1.87	82.25-89.68
730	79.56	17.73	1.78	76.02-83.09
1,095	74.47	17.12	1.72	71.06-77.89

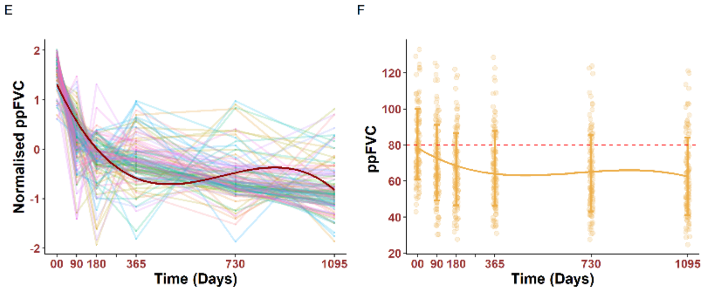

Cluster 3 - n=111				
**Days**	**ppFVC**	**sd**	**sem**	**95% CI**
1	80.58	19.54	1.86	76.89-84.28
90	70.20	20.95	2.00	66.25-74.16
180	66.60	20.07	1.91	62.81-70.4
365	66.96	20.73	1.98	63.04-70.87
730	64.31	21.08	2.01	60.33-68.29
1,095	62.67	21.46	2.05	58.62-66.73

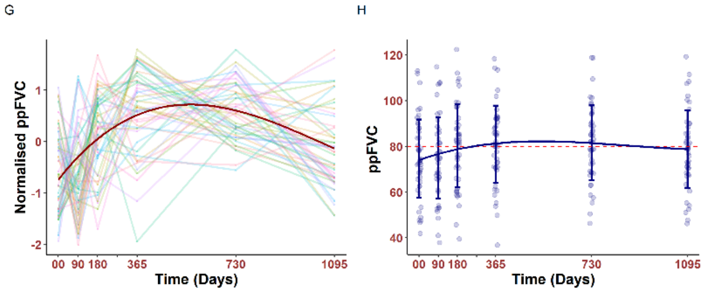

Cluster 4 - n=45				
**Days**	**ppFVC**	**sd**	**sem**	**95% CI**
1	74.63	17.13	2.58	69.42-79.83
90	74.90	17.83	2.69	69.48-80.31
180	80.38	18.21	2.75	74.84-85.91
365	80.85	16.97	2.56	75.69-86
730	81.51	16.44	2.48	76.51-86.5
1,095	78.77	17.12	2.58	73.57-83.98

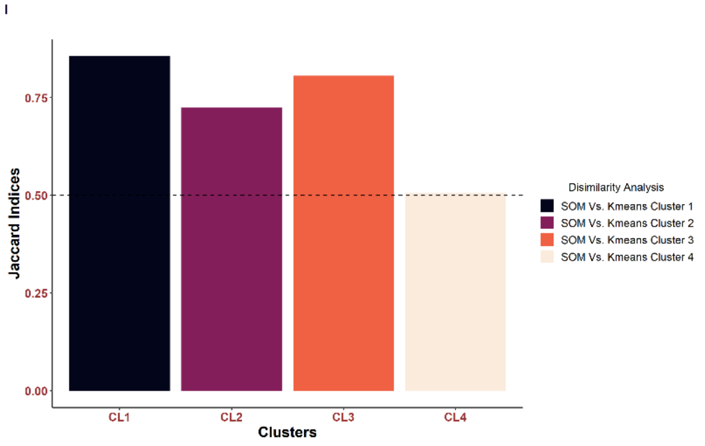

Suppl. Table 1

	PROFILE(n=415)	Survivors>3Yr(n=242)	No missing data(n=82)	Replication(n=180)
Percentage (%)	100.00	58.31	19.76	100
Age	70.56±7.88	70.45±7.65	71.87±6.5	68.44±8.09 *
Men (%)	77.35	74.79	78.05	78.33
Ever Smoker (%)	71.81	74.38	74.39	65.56 A*
Never Smoker (%)	28.19	25.62	25.61	32.22 A
ppFVC Baseline	80.12±19.23	85.38±18.6 **	89.23±19.78 **	69.19±16.25 **
ppDLco Baseline	46.03±14.98 A	50.87±15.13 B**	53.06±14.84 C**	
ppFEV1 Baseline	82.7±18.06 A	86.98±17.63 **	90.07±17.72 **	
< 5-year survival (% patients)	35.90	61.57	65.85	24.44

Suppl. Table 2

Spirometric Appoments	Expected spirometric visits time Frame PROFILE (Days)	Range in days for visit hamonisation in the Chicago dataset
Baseline	0	0
Visit 2	90	70-177
Visit 3	180	154-350
Visit 4	365	322-581
Visit 5	730	564-823
Visit 6	1095	1017-1251

Suppl. Table 3

Cluster 1								
Chr.	Position	rsid	Locus	EAF in Cluster	EAF in other clusters	OR	95% CI	P-Value
3	169,486,271	rs9860874	TERC	33.30%	32.40%	1.04	[0.74, 1.47]	0.781
3	44,903,434	rs2292181	KIF15	8.60%	6.30%	1.39	[0.76, 2.55]	0.142
4	89,837,808	rs2609259	FAM13A	27.90%	25.90%	0.90	[0.63, 1.30]	0.646
5	1,282,414	rs7725218	TERT	27.00%	28.40%	0.93	[0.65, 1.34]	0.541
5	169,015,479	rs116483731	SPDL1	2.70%	2.50%	1.09	[0.40, 3.00]	0.657
6	7,563,232	rs2076295	DSP	48.60%	41.40%	0.75	[0.54, 1.03]	0.086
7	99,630,342	rs2897075	ZKSCAN1	40.50%	45.50%	0.82	[0.59, 1.13]	0.326
7	1,868,761	rs12537430	MAD1L1	34.70%	33.60%	0.95	[0.68, 1.34]	0.615
8	120,940,206	rs10808505	DEPTOR	37.40%	40.50%	0.88	[0.63, 1.22]	0.396
10	111,229,861	rs79684490	10q25.1	6.30%	4.70%	1.36	[0.68, 2.72]	0.427
11	1,241,221	rs35705950	MUC5B	37.80%	32.70%	1.26	[0.90, 1.76]	0.129
13	113,534,984	rs9577395	ATP11A	14.90%	17.60%	0.82	[0.53, 1.28]	0.404
15	86,287,910	rs11073517	AKAP13	29.30%	37.20%	0.70	[0.49, 0.99]	0.022
15	40,931,708	rs12912339	KNL1	19.40%	19.10%	0.99	[0.66, 1.48]	0.816
15	40,716,253	rs2304645	IVD	42.80%	43.20%	1.02	[0.74, 1.41]	0.939
16	162,240	rs74614704	NPRL3	7.70%	7.20%	1.07	[0.58, 1.97]	0.974
17	44,214,888	rs2077551	17q21.31	16.70%	15.50%	1.09	[0.70, 1.68]	0.515
19	4,717,672	rs12610495	DPP9	36.50%	34.20%	1.10	[0.79, 1.54]	0.388
20	62,284,170	rs112087793	STMN3	6.80%	7.00%	0.97	[0.51, 1.83]	0.889
20	62,324,391	rs41308092	RTEL1	5.00%	2.90%	1.73	[0.76, 3.92]	0.183

Cluster 2								
Chr.	Position	rsid	Locus	EAF in Cluster	EAF in other clusters	OR	95% CI	P-Value
3	169,486,271	rs9860874	TERC	32.90%	32.70%	1.01	[0.69, 1.48]	0.778
3	44,903,434	rs2292181	KIF15	7.00%	7.10%	0.98	[0.49, 1.98]	0.946
4	89,837,808	rs2609259	FAM13A	27.80%	26.20%	0.92	[0.62, 1.37]	0.509
5	1,282,414	rs7725218	TERT	26.60%	28.30%	0.92	[0.61, 1.37]	0.657
5	169,015,479	rs116483731	SPDL1	2.50%	2.60%	0.99	[0.32, 3.08]	0.821
6	7,563,232	rs2076295	DSP	38.60%	45.50%	1.33	[0.92, 1.91]	0.166
7	99,630,342	rs2897075	ZKSCAN1	43.00%	44.10%	0.96	[0.67, 1.37]	0.690
7	1,868,761	rs12537430	MAD1L1	34.80%	33.70%	0.95	[0.65, 1.38]	0.685
8	120,940,206	rs10808505	DEPTOR	40.50%	39.20%	1.06	[0.73, 1.52]	0.881
10	111,229,861	rs79684490	10q25.1	7.00%	4.70%	1.51	[0.72, 3.15]	0.211
11	1,241,221	rs35705950	MUC5B	36.70%	33.70%	1.14	[0.79, 1.66]	0.665
13	113,534,984	rs9577395	ATP11A	17.70%	16.30%	1.10	[0.69, 1.77]	0.816
15	86,287,910	rs11073517	AKAP13	38.00%	33.50%	1.22	[0.84, 1.76]	0.188
15	40,931,708	rs12912339	KNL1	17.10%	19.90%	1.20	[0.75, 1.92]	0.456
15	40,716,253	rs2304645	IVD	41.80%	43.50%	1.07	[0.75, 1.54]	0.653
16	162,240	rs74614704	NPRL3	10.10%	6.50%	1.62	[0.87, 3.03]	0.100
17	44,214,888	rs2077551	17q21.31	17.70%	15.40%	1.19	[0.74, 1.91]	0.881
19	4,717,672	rs12610495	DPP9	29.70%	36.60%	0.73	[0.50, 1.08]	0.207
20	62,284,170	rs112087793	STMN3	7.00%	6.90%	1.01	[0.50, 2.04]	0.909
20	62,324,391	rs41308092	RTEL1	3.20%	3.70%	0.84	[0.31, 2.29]	0.647

Cluster 3								
Chr.	Position	rsid	Locus	EAF in Cluster	EAF in other clusters	OR	95% CI	P-Value
3	169,486,271	rs9860874	TERC	29.00%	34.20%	0.79	[0.55, 1.14]	0.309
3	44,903,434	rs2292181	KIF15	6.50%	7.30%	0.88	[0.44, 1.73]	0.451
4	89,837,808	rs2609259	FAM13A	29.00%	25.60%	0.84	[0.58, 1.23]	0.404
5	1,282,414	rs7725218	TERT	30.10%	27.10%	1.16	[0.80, 1.68]	0.376
5	169,015,479	rs116483731	SPDL1	3.80%	2.10%	1.84	[0.69, 4.90]	0.270
6	7,563,232	rs2076295	DSP	41.90%	44.60%	1.11	[0.79, 1.57]	0.549
7	99,630,342	rs2897075	ZKSCAN1	34.40%	33.80%	0.97	[0.68, 1.39]	0.771
7	1,868,761	rs12537430	MAD1L1	47.30%	42.50%	1.21	[0.86, 1.71]	0.310
8	120,940,206	rs10808505	DEPTOR	34.90%	41.30%	0.77	[0.54, 1.09]	0.158
10	111,229,861	rs79684490	10q25.1	3.20%	6.00%	0.52	[0.21, 1.27]	0.098
11	1,241,221	rs35705950	MUC5B	30.10%	36.00%	0.76	[0.53, 1.10]	0.234
13	113,534,984	rs9577395	ATP11A	17.20%	16.50%	1.05	[0.67, 1.66]	0.691
15	86,287,910	rs11073517	AKAP13	21.50%	18.30%	0.82	[0.54, 1.25]	0.537
15	40,931,708	rs12912339	KNL1	46.20%	41.90%	0.84	[0.60, 1.18]	0.362
15	40,716,253	rs2304645	IVD	32.30%	35.40%	0.87	[0.61, 1.24]	0.318
16	162,240	rs74614704	NPRL3	4.80%	8.30%	0.56	[0.27, 1.18]	0.101
17	44,214,888	rs2077551	17q21.31	16.70%	15.60%	1.08	[0.68, 1.71]	0.450
19	4,717,672	rs12610495	DPP9	36.60%	34.40%	1.10	[0.77, 1.57]	0.967
20	62,284,170	rs112087793	STMN3	1.60%	4.40%	0.36	[0.11, 1.22]	0.050
20	62,324,391	rs41308092	RTEL1	6.50%	7.10%	0.90	[0.46, 1.79]	0.635

Cluster 4								
Chr.	Position	rsid	Locus	EAF in Cluster	EAF in other clusters	OR	95% CI	P-Value
3	169,486,271	rs9860874	TERC	38.00%	31.80%	1.31	[0.85, 2.04]	0.216
3	44,903,434	rs2292181	KIF15	5.00%	7.40%	0.66	[0.25, 1.70]	0.339
4	89,837,808	rs2609259	FAM13A	17.00%	28.30%	1.92	[1.11, 3.34]	0.013
5	1,282,414	rs7725218	TERT	28.00%	27.90%	1	[0.63, 1.61]	0.802
5	169,015,479	rs116483731	SPDL1	0.00%	3.00%	NA		0.019
6	7,563,232	rs2076295	DSP	45.00%	43.60%	0.95	[0.62, 1.45]	0.846
7	99,630,342	rs2897075	ZKSCAN1	30.00%	34.60%	1.24	[0.78, 1.96]	0.140
7	1,868,761	rs12537430	MAD1L1	46.00%	43.50%	1.11	[0.72, 1.70]	0.619
8	120,940,206	rs10808505	DEPTOR	51.00%	37.50%	1.74	[1.13, 2.66]	0.009
10	111,229,861	rs79684490	10q25.1	4.00%	5.50%	0.72	[0.25, 2.08]	0.562
11	1,241,221	rs35705950	MUC5B	31.00%	35.00%	0.84	[0.53, 1.32]	0.325
13	113,534,984	rs9577395	ATP11A	18.00%	16.40%	1.12	[0.64, 1.95]	0.780
15	86,287,910	rs11073517	AKAP13	18.00%	19.40%	1.1	[0.63, 1.91]	0.835
15	40,931,708	rs12912339	KNL1	40.00%	43.60%	1.16	[0.75, 1.79]	0.498
15	40,716,253	rs2304645	IVD	45.00%	32.70%	1.69	[1.09, 2.59]	0.008
16	162,240	rs74614704	NPRL3	7.00%	7.40%	0.94	[0.41, 2.15]	0.947
17	44,214,888	rs2077551	17q21.31	10.00%	17.00%	0.54	[0.27, 1.08]	0.638
19	4,717,672	rs12610495	DPP9	37.00%	34.60%	1.11	[0.71, 1.72]	0.775
20	62,284,170	rs112087793	STMN3	5.00%	3.40%	1.52	[0.55, 4.16]	0.323
20	62,324,391	rs41308092	RTEL1	8.00%	6.70%	1.21	[0.55, 2.67]	0.693

Legends

Suppl. fig 1: Graph A) Column graphs represent the performance of six imputation methods after imputing simulated missing data. Each colour column represents a different spirometry visit. Methods of imputation used were FVC decline 10% per year (-10%DPY), K Nearest Neighbour (KNN), Last Observation Carried Forward (LOCF), Linear Regression (LR), Random Forest (RF) and Random Forest with Markov Chain Monte Carlo adjustment (RF-MCMC). Each horizontal dotted line indicates the best mean NRMSD recorded performance on each visit. The affect of imputed values on the mean ppFVC on the training dataset was plotted for each imputation method (B-G). The green line illustrates mean trend and dots reflect actual values in each plot. B) Grey dots and line represent RF imputation. C) Red dots and line represent KNN imputation. D) Dark blue dots and line represent LOCF imputation. E) Yellow dots and line represent -10%DPY imputation. F) Light blue dots and line represent LR imputation. G) Grey dots and line represent MCMC_RF imputation.

Suppl. fig 2: Sensitivity analysis on imputation by RF trained by Naïve or Theoretical MCMC synthetic databases. In each graph the red columns represent the NRMSD index error in imputation on spirometric visits 3 to 7 by RF A) trained by the MCMC naïve synthetic database or B) Theoretical database. Graph C The dots shown are for patients or synthetic values with available data without imputation (red) as well as for the results of two imputation methods used to account for missing data (naïve- yellow and theoretical -green). The lines represent the mean values non-imputed (red) and imputed (yellow and green). Boxplot illustrates mean (±SE) changes from baseline.

Suppl. fig 3: Cluster sensitivity and validity analysis one. Graphs A to D, within-Sum of Square inertia (or variance) graphs for the cluster data partition generated by the elbow algorithm, from 1 to 10 clusters for the whole PROFILE lung function cohort. Each graph represents a different lung function data partition: A) 1095 days, B) 730 days, C) 360 days or D) 1095 days data of not imputed and complete spirometric records. The estimated curvature is displayed as a dashed solid line and shows in each data partition case a stabilisation decline (or inertia) at 4 clusters indicated by a dashed vertical line. E) Jaccard indices for each data partition, the columns are the value obtained by each iteration, every six columns represent the stability of a cluster, the vertical red dashed line represents the minimum stability threshold.

Suppl. fig 4: Second Cluster sensitivity and validity analysis. Graphs A to C Within-Sum of Square inertia (or variance) graphs for the cluster data partition generated by the elbow algorithm, from 1 to 10 clusters for the PROFILE lung function cohort. Each graph represents a different lung function data partition: A) lung function from the whole PROFILE cohort (n=415), B) only on patients who missed spirometric visits not due to death (n=242), or C) Only patients who completed all the spirometric visits without imputation (n=82). The estimated curvature is displayed as a dashed solid line and shows in each data partition case a stabilisation decline (or inertia) at 4 clusters indicated by a dashed vertical line. D) Jaccard indices for each data partition, the columns are the value obtained by each iteration, every three columns represent the stability of a cluster, the vertical red dashed line represents the minimum stability threshold.

Suppl. fig 5: Graphs A to D represent the mean ppFVC for training dataset (green line), survivors' dataset (yellow line) and complete PROFILE cohort (red line) stratified at (A) CL1, (B) CL2, (D) CL3 and (D) CL4. The table below each graph are results obtained by the cluster based linear mixed model analysis. For fixed effects, the full model includes the continuous variable of visits FVC and time of visit. Each result was contrasted by the training database. The values were centred by z-score transformation of raw score to overcome the problem of collinearity. Significance of each model is indicated by ∗p-value < 0.05; ∗∗p-value < 0.01; ∗∗∗p-value < 0.001. visit SP (1) survivors contrasted by training dataset – visit FP (1) complete database contrasted by training dataset.

Suppl. fig 6: Within-Sum of Square inertia (or variance) graphs for the cluster data partition generated by the elbow algorithm, from 1 to 10 clusters for the whole Chicago Consortium lung function cohort. Each graph represents a different lung function data partition: A) 1095 days, B) 730 days, C) 360 days or D) 1095 days data of not imputed and complete spirometric records. The estimated curvature is displayed as a dashed solid line and shows in each data partition case a stabilisation decline (or inertia) at 4 or 5 clusters indicated by a dashed vertical line. Average lung functions of the four clusters obtained from Self Organising Maps of three years of spirometry data from patients recruited by the Chicago Consortium. Solid colour lines in each graph represent the mean trajectory ppFVC. Dots: The actual ppFVC values of each patient. Each box blot represents mean (±SE) changes on each time point on

the same colour patterns as previously described. Cluster E) CL1, n=26 - green, F) CL2, n= 42 - red, G) CL4, n= 38 - yellow and H) CL4, n= 74 - blue.

Suppl. fig 7: Kaplan-Meir survival graphs (Chicago). Kaplan-Meier estimates of survival of IPF patients based on A) cluster allocation. B) the number of deaths is shown for every cluster during the following five years.

Suppl. fig 8: Graphs A to D: raw KM graphs and COX proportional hazards of each cluster per study site. In all graphs, the green colour represents PROFILE and red represents the data from the Chicago (Replication) cohort. The tables on the right of each KM graphs represent the mortality hazard ratio (HR) from each cohort observed in each cluster. The values recorded in the top two rows of each table are raw HR, and the subsequent two values represent the FVC adjusted HR values. The Cox proportional hazard regression model used for this analysis assumes the following relationship: h_0 is called the baseline hazard (=0), *h(t)* represents the survival time, the coefficient (cluster allocation and baseline ppFVC registered in each study – PROFILE and Chicago cohorts – b1, b2) is the impact of and size of each covariant.

$$h(t) = h_0 \times \exp(b_1x_1 + b_2x_2 + \cdots + b_px_p)$$

Suppl. fig 9: Baseline comparison of biomarker concentrations in the four clusters of patients with IPF in the PROFILE cohort. Median plus IQR shown. Dots: The values of actual biomarkers observed in each patient – A) C3M - CL1 n=125, CL2 n=87, CL3 n=102, CL4 n=59. B) C6M - CL1 n=125, CL2 n=86, CL3 n=102, CL4 n=59. C) PRO C3 - CL1 n=127, CL2 n=88, CL3 n=102, CL4 n=59. D) PRO C6 - CL1 n=127, CL2 n=88, CL3 n=102, CL4 n=59. E) cyfra211- CL1 n=129, CL2 n=89, CL3 n=100, CL4 n=58. F) MMP7- CL1 n=83, CL2 n=63, CL3 n=75, CL4 n=38.

Suppl. fig 10: K-means derived clusters of FVC trajectory in PROFILE study population. Individual dots indicate the values obtained by the naïve RF-MCMC model on each patient. Trendline illustrates mean± IQR ppFVC for each time point. The graphs A, C, E, G represent each individual spirometry traces

clustered by SOM from each patient represented as scale-free units normalised values. Graphs B, D, F and H represent each cluster of patients and the data is represented as not normalised ppFVC. Graphs: A-B) Classical linear decline (CL1, n=159 - green), C-D) Initial increase trajectory (CL2, n=100-red), E-F) Early decline trajectory (CL3, n=111 - yellow) and G-H) Stable trajectory (CL4, n=45 - blue). Tables indicate descriptive statistics for each cluster. ppFVC -percentage predicted forced vital capacity at each time point. SD - standard deviation. 95% CI confidence interval upper and lower bound. I) Jaccard indices for each data partition generated by the K-means and SOM algorithms. The columns are the value obtained by each clustering method; the heights of each of the four columns represent the stability of a cluster and the vertical red dashed line represents the minimum stability threshold.

Suppl. Table 1: Comparison of baseline clinical characteristics between datasets used in this study. Data are mean±SD or n (%), unless otherwise stated. ppFVC – percent predicted forced vital capacity; ppDLco - percent predicted diffusion capacity for carbon monoxide; ppFEV1 - percent predicted forced expiratory volume in 1 second. Number of patients analysed due to missing data ppDLco: A=380, B=260, C=76; ppFEV1 A=413. Statistically significant differences denoted by *p-value<0.005; **p-value<0.001 (Bonferroni adjusted).

Suppl. Table 2: Parameters of the visit time frame harmonization between PROFILE and Chicago (Independent) Cohorts.

Suppl. Table 3: Summary statistics of the comparison of individuals in a single cluster with individuals in the other three clusters from each GWAS for the 20 SNPs that have been previously identified as being genome-wide associated with IPF susceptibility by Allen et al 2019 (4). Definition of abbreviations: Chr = chromosome; CI = confidence interval; rsid = reference SNP cluster ID; EAF = effect allele frequency; SE = standard error; OR= Odd ratios.

References

1. Jenkins RG, Simpson JK, Saini G, et al. Longitudinal change in collagen degradation biomarkers in idiopathic pulmonary fibrosis: an analysis from the prospective, multicentre PROFILE study. Lancet Respir Med. 2015;3(6):462-72.
2. Noth I, Zhang Y, Ma SF, et al. Genetic variants associated with idiopathic pulmonary fibrosis susceptibility and mortality: a genome-wide association study. Lancet Respir Med. 2013;1(4):309-17.
3. Vesanto J, Alhoniemi E. Clustering of the self-organizing map. IEEE Trans Neural Netw. 2000;11(3):586-600.
4. Allen RJ, Guillen-Guio B, Oldham JM, et al. Genome-Wide Association Study of Susceptibility to Idiopathic Pulmonary Fibrosis. Am J Respir Crit Care Med. 2020;201(5):564-74.
